# Long-Range Gene Flow and the Effects of Climatic and Ecological Factors on Genetic Structuring in a Large, Solitary Carnivore: The Eurasian Lynx

**DOI:** 10.1371/journal.pone.0115160

**Published:** 2014-12-31

**Authors:** Mirosław Ratkiewicz, Maciej Matosiuk, Alexander P. Saveljev, Vadim Sidorovich, Janis Ozolins, Peep Männil, Linas Balciauskas, Ilpo Kojola, Henryk Okarma, Rafał Kowalczyk, Krzysztof Schmidt

**Affiliations:** 1 Institute of Biology, University of Białystok, Białystok, Poland; 2 B. M. Zhitkov Russian Research Institute of Game Management and Fur Farming, Russian Academy of Sciences, Kirov, Russia; 3 Centre for Biological Resources (former Institute of Zoology) of National Academy of Sciences, Minsk, Belarus; 4 Latvian State Forest Research Institute “Silava”, Salaspils, Latvia; 5 Estonian Environment Agency, Tartu, Estonia; 6 Nature Research Centre, Vilnius, Lithuania; 7 Finnish Game and Fisheries Research Institute, Oulu Game and Fisheries Research, University of Oulu, Finland; 8 Institute of Nature Conservation, Polish Academy of Sciences, Kraków, Poland; 9 Mammal Research Institute, Polish Academy of Sciences, Białowieża, Poland; University of York, United Kingdom

## Abstract

Due to their high mobility, large terrestrial predators are potentially capable of maintaining high connectivity, and therefore low genetic differentiation among populations. However, previous molecular studies have provided contradictory findings in relation to this. To elucidate patterns of genetic structure in large carnivores, we studied the genetic variability of the Eurasian lynx, *Lynx lynx* throughout north-eastern Europe using microsatellite, mitochondrial DNA control region and Y chromosome-linked markers. Using SAMOVA we found analogous patterns of genetic structure based on both mtDNA and microsatellites, which coincided with a relatively little evidence for male-biased dispersal. No polymorphism for the cytochrome *b* and *ATP6* mtDNA genes and Y chromosome-linked markers were found. Lynx inhabiting a large area encompassing Finland, the Baltic countries and western Russia formed a single genetic unit, while some marginal populations were clearly divergent from others. The existence of a migration corridor was suggested to correspond with distribution of continuous forest cover. The lowest variability (in both markers) was found in lynx from Norway and Białowieża Primeval Forest (BPF), which coincided with a recent demographic bottleneck (Norway) or high habitat fragmentation (BPF). The Carpathian population, being monomorphic for the control region, showed relatively high microsatellite diversity, suggesting the effect of a past bottleneck (e.g. during Last Glacial Maximum) on its present genetic composition. Genetic structuring for the mtDNA control region was best explained by latitude and snow cover depth. Microsatellite structuring correlated with the lynx's main prey, especially the proportion of red deer (*Cervus elaphus*) in its diet. Eurasian lynx are capable of maintaining panmictic populations across eastern Europe unless they are severely limited by habitat continuity or a reduction in numbers. Different correlations of mtDNA and microsatellite population divergence patterns with climatic and ecological factors may suggest separate selective pressures acting on males and females in this solitary carnivore.

## Introduction

Various studies on population genetics in large carnivores show diverse patterns of genetic structure. Some research emphasizes that, due to their high mobility, carnivore populations are maintained through long distance dispersal, which contributes to high gene flow across large spaces and low differentiation among populations [1]–[6]. There is also an increasing number of studies showing that gene flow does not occur equally in different directions, indicating the existence of various barriers for effective dispersal, including both physical obstacles and ecological conditions [7]–[16]. Genetic differentiation among carnivore populations has also been found to be related with the phylogeographic histories of species such as brown bear [17], [18] or wolf [19], [20].

Some of these discrepancies may result from using markers of different heritability, e.g. microsatellites and mitochondrial DNA (mtDNA) – the markers most frequently used in population genetic studies. For instance, a microsatellite-based study on the genetic structure of Scandinavian brown bears [21] found no support for the pattern of population subdivision previously based on mtDNA [22]. While mtDNA can be used to resolve taxonomy, questions on historical genetic variation and population structure, microsatellite markers are more suitable for inferring recent population history and contemporary gene flow [23].

From the research concerning the population genetic structure of the Eurasian lynx (*Lynx lynx*), two studies [24], [25] included both microsatellites and mtDNA. While Hellborg et al. [24] focused on understanding the causes of genetic differentiation within the Scandinavian and Baltic populations of lynx, the most recent study by Rueness et al. [25] covered almost the entire range of the species. This largest-scale study to date of spatial genetic patterns of the Eurasian lynx [25] has revealed a pronounced pattern of genetic structuring along the west-east axis of Eurasia, though the authors admitted the necessity of more fine-scaled sampling to account for the variability of ecological factors to explain the observed restrictions in gene flow. A previous study by Ratkiewicz et al. [26] based on the mtDNA control region, sampled the western edge of the species range (including the area between Norway and the Carpathian Mountains) and identified the existence of four genetic units that could have resulted from the marginal location of the populations studied, habitat fragmentation and/or demography. However, using a single genetic marker was not sufficient to assess the relative importance of these different factors. The importance of climatic factors on lynx genetic differentiation has been recently suggested by a review study [12]. Moreover, the Eurasian lynx was shown to shift its diet throughout its vast range with lynx in more northerly locations preying mainly on hares (*Lepus timidus*) (Russia, Finland) or domestic reindeer (*Rangifer tarandus*) (Norway) and the south-western populations feeding on Cervids [27]. This could potentially contribute to genetic divergence among these populations through local adaptations.

The aim of our study was to investigate the factors contributing to the patterns of genetic structuring in Eurasian lynx. To achieve this objective, we intensively sampled a large portion of the lynx's range (including sites at the periphery and within the core area of the species' distribution) and used three types of genetic markers: maternally inherited mtDNA, Y-chromosome markers and nuclear microsatellites. Our primary goal was to understand the relative role of factors affecting the genetic diversity in different populations. We predicted that: 1) lynx are capable of maintaining high rates of gene exchange providing that habitat is continuous; 2) genetic variability and distinctiveness of contemporary lynx populations in Eastern Europe are affected by both human activity (habitat fragmentation, population extermination) and past climatic events (Last Glacial Maximum); 3) genetic differentiation between lynx populations is correlated with climatic factors and/or 4) it is correlated with the main prey type of lynx. This knowledge will help in interpreting the importance of current and historical processes in shaping the distribution and genetic structure of the lynx population, as a model species of large, mobile carnivores.

## Material and Methods

### Ethics statement

The license for lynx live-trapping and blood sampling was obtained from the National Ethics Committee for Animal Experiments (no: DB/KKE/PL – 110/2001) and the Local Ethics Committee for Animal Experiments at the Medical University of Białystok (no: 52/2007). No animals were harmed during live-trapping and handling. An effort was made to minimize the time animals spent immobilized in the traps. All traps were equipped with radio- or GSM-alarm systems that allowed us to remotely control trapping and release the captured animals from the traps within 15 minutes to 1 hour.

### Sampling

**Figure 1:**
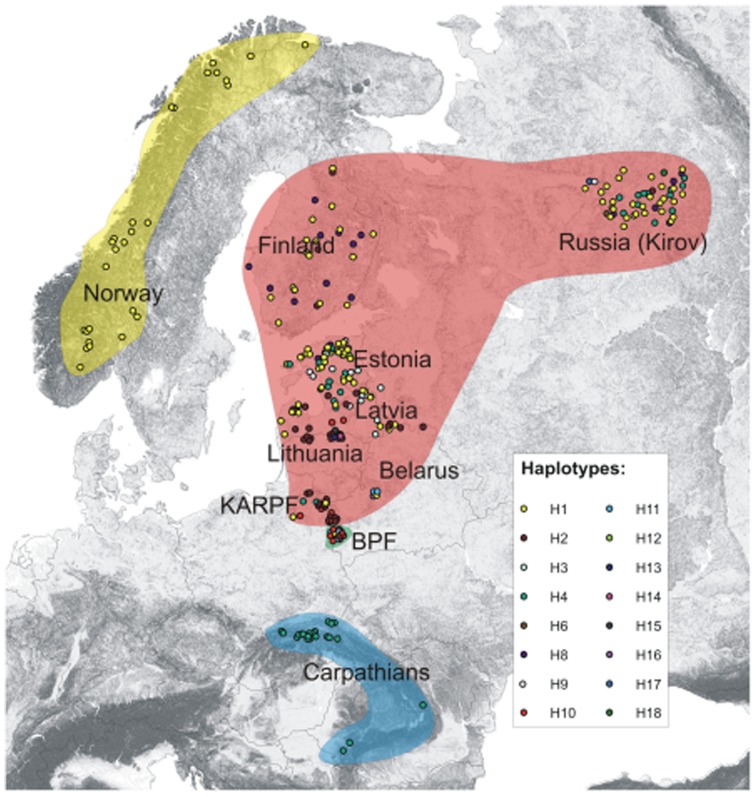
Distribution of Eurasian lynx sampling locations and mtDNA haplotypes. Map showing distribution of sampling locations of the Eurasian lynx in north-eastern and central Europe. The color of each sampled individual denotes the haplotype of cr mtDNA and corresponds to the haplotype network in Fig. 2. Points are clustered into four groups as assigned by SAMOVA (both based on mtDNA and microsatellites) and shaded with different colors: Norway (yellow), BPF (green), Carpathians (blue) and remaining samples (pink). Names of arbitrarily assigned populations are given. Intensity of grey shading refers to the terrain ruggedness indicating mountainous areas. The background map was extracted from open access database available through USGS: http://srtm.usgs.gov/index.php). It is similar but not identical to the original image, and is therefore for representative purposes only.

We analysed samples of Eurasian lynx from northern, central and eastern Europe bordered by Norway, the Carpathian Mountains and Russia (Kirov Republic) (Table 1). We arbitrarily assigned them to ten populations according to their geographic location and country of origin (Fig. 1). We are thus using the term “population” for simplicity, although we acknowledge that they may not represent populations in an ecological sense. One should note that Lithuanian lynxes were mostly sampled near to the Latvian boundary, while Belarusian lynxes were mostly sampled close to the Latvian and Russian borders (Fig. 1). Sampled lynxes span an area large enough to include a variety of habitat and climatic conditions. There is also a significant dietary diversification among the lynx populations over the sampled area (see S4 Table and references therein).

**Table 1:** Mitochondrial DNA- control region diversity indices for the Eurasian lynx samples studied.

No	Population	*N*	*Nh*	*h* (±SE)	*π* (±SE)	*S*	PD
1.	Norway*	30	1	0.00 (±0.00)	0.00 (±0.00)	0	0 (±0.00)
2.	Finland*	29	3	0.62 (±0.05)	0.16 (±0.12)	3	0.96(±0.67)
3.	Estonia#	58	6	0.70 (±0.05)	0.35 (±0.22)	8	2.15 (±1.21)
4.	Latvia#	47	7	0.81 (±0.03)	0.47 (±0.29)	11	2.29 (±1.28)
5.	Lithuania	14	4	0.40 (±0.16)	0.07 (±0.08)	3	0.43 (±0.41)
6.	Belarus	11	5	0.82 (±0.08)	0.44 (±0.29)	8	2.69 (±1.55)
7.	Poland# (KARPF)	26	4	0.64 (±0.07)	0.29 (±0.20)	6	1.80 (±1.07)
8.	Poland# (BPF)	25	4	0.36 (±0.12)	0.14 (±0.11)	6	0.84 (±0.62)
9.	Carpathians#	40	1	0.00 (±0.00)	0.00 (±0.00)	0	0 (±0.0)
10.	Russia (Kirov)	52	8	0.64 (±0.06)	0.25 (±0.17)	7	1.56 (±0.94)
	All	332	16	0.78 (±0.01)	0.388 (±0.23)	18	2.38 (±1.30)

*N* – sample size, *Nh* – number of haplotypes, *h* – haplotype diversity, *π* – nucleotide diversity (%), *S* – number of segregating sites, PD – mean number of pairwise differences, SE – standard error, KARPF – Knyszyn, Augustów, Rominta, Piska Forests, BPF – Białowieża Primeval Forest. * - data from Ratkiewicz et al. [30], # - increased sample size from data utilized in Ratkiewicz et al. [30].

Altogether, 332 lynx individuals were sampled between 1992 and 2011. Sample sizes per population are given in Table 1. Samples were tissue collected from legally hunted individuals (Norway, Finland, Estonia, Latvia, Russia and Romania; n = 216), blood of live-trapped lynx (Białowieża Primeval Forest (BPF) in NE Poland; n = 18), hair samples (n = 54), animals found dead (Belarus, Lithuania, Poland (n = 25) and museum specimens (Poland, Slovakia, Ukraine; n = 19). Hairs were sampled non-invasively based on methods described by Schmidt and Kowalczyk [28]. Samples from northeastern Poland were assigned to two separate subpopulations (BPF and KARPF (Knyszyn, Augustów, Rominta, Piska Forests); Table 1) based on previous mtDNA results [26]. The Carpathian population consisted of samples from Poland, Slovakia, Romania and Ukraine which were pooled together due to the high geographic isolation of the Carpathian Mountains from the other populations in the distribution range and because only a single mtDNA haplotype shared by all individuals in this area (see Results).

### DNA extraction, microsatellite amplification and error checking

Genomic DNA was extracted using the Genomic Mini Kit (A&A Biotechnology, Gdynia, Poland) according to the manufacturer's protocol and was stored at −20°C until used. The amplification of 13 microsatellite markers (twelve nuclear and one Y-linked) was carried out in three multiplex panels (Panel 1: Fca045, Fca090, Fca149, Fca391 and Fca559; Panel 2: Fca001, Fca008, Fca031, Fca043, F115; Panel 3: Fca077, Fca078 and Y-linked 278g21-4). The above-mentioned microsatellite loci were developed for domestic cats [29], [30] and successfully used in several lynx studies: [24], [25], [28], [31], [32]. The final reaction volume was 10 µl. Mastermix was created for 8-sample batches and included 36 µl Qiagen Multiplex Master Mix, 21 µl ultra-pure H_2_O (Qiagen, Hilden, Germany), 7 µl primer mix (containing 2pmol of each primer). Aliquots of 8 µl of this mix was then added to 2 µl of isolated DNA template. The thermocycling profile was as follows: 95°C for 15 min, followed by 30–45 cycles of denaturation at 94°C for 30 s, annealing at 57°C (Panel 1 and 3) or 56.5°C (Panel 2) for 90 s, extension at 72°C for 60 s and final extension at 60°C for 30 min. The PCR products were mixed with 10 µl ultragrade formamide and 0.2 µl GeneScan 500-LIZ size standard (Life Technologies, Carlsbad, CA, USA), denatured at 95°C for 5 min, immediately cooled and then separated using a four-capillary ABI 3130 Genetic Analyser (Life Technologies, Carlsbad, CA, USA). Allele sizes and genotypes were scored using GeneMapper 4.0 software (Life Technologies, Carlsbad, CA, USA). PCRs were repeated twice to test the consistency of the results for a subset (5%) of randomly chosen individuals. A total of 54 hair samples from Lithuania and Belarus were analyzed. Hair samples which gave low amplification rate and displaying contamination within hair traps were excluded. Microsatellite panels in the remaining hair samples (20) were amplified between two and four times until they gave reliable genotypes, e.g. heterozygotes at least three times out of four. Samples with inconsistent genotypes were discarded from the analysis. Possible genotyping errors due to stuttering, short allele dominance and null alleles were tested by MICRO-CHECKER 2.2.3. [33]. Multilocus genotypes of the hair samples were analysed in CERVUS 3.0.3 [34] to avoid using samples of the same individuals. Obtained genotypes are presented in S1 Appendix.

### mtDNA and Y-chromosome analyses

A portion (613 bp) of the mtDNA control region (cr mtDNA) was amplified in 122 lynxes using the primer pair LGL283 and ISM015 [26], [35]. Additionally, 210 lynx cr mtDNA sequences from Ratkiewicz et al. [26] were used. Almost the entire cytochrome b gene, e.g. 1094 bp (mtDNA) was amplified for 12 individuals from Russia, Finland, Estonia, Latvia and Poland using lynx-specific primers: LcytbF: 5′ -CAC ATG GAA TTT AAC CAT GAC C -3′ and LcytbR: 5′-GAC TCT TCA TTT GAG GAG ACG. The entire 681 bp of the ATP6 gene (LATP_F: 5′-TCC AGA ACC TAA ATC CAC AAC C-3′ and LATP_R: 5′-GCA TGA GTT TGG TGG GTC ATT A-3′) was amplified in 8 lynxes from Norway, Finland, Latvia and Poland. All laboratory procedures and quality test controls for cr mtDNA followed [26]. We also amplified seven universal Y-linked markers (YCATS, Y-chromosome conserved anchor tagged sequences) according to [36], with the exception of marker *DBY4*, which had an annealing temperature of 48°C. We obtained clear PCR products for *DBY4* (185 bp), *DBY7* (275 bp), *DBY8* (115 bp), *SMCY7* (625 bp) and *SMCY17* (125 bp) but no clear products were obtained for *UBE1Y6* and *UTY11*. The five Y-chromosome markers that gave clear PCR products were sequenced in 43 males from Russia, Finland, Estonia, Latvia, Poland, Romania and Belarus according to [36].

### Statistical analyses

For the autosomal microsatellite loci, FSTAT 2.9.3 [37] was used to estimate the number of alleles per locus (N_A_), allelic richness (A_R_), gene diversity (Hs) and inbreeding coefficient (*F*_IS_) in the populations. Differences among groups of samples in A_R_, Hs, expected heterozygosity (H*e*), relatedness coefficient (r), and *F*_ST_ were tested using a permutation procedure (10000 iterations) in FSTAT. The same software was used to tests for linkage disequilibrium among 12 microsatellite loci within each population and for the total sample (13200 permutations). Hardy–Weinberg equilibrium probabilities were calculated using GENEPOP 4.0 [38]. Estimates of cr mtDNA haplotype diversity (h) and nucleotide diversity (*π*), number of segregating sites (s) and mean pairwise difference were calculated using ARLEQUIN v 3.1 [39]. Relationships among haplotypes were represented as a haplotype network obtained with the statistical parsimony method using the TCS v 1.21software [40]. Pairwise *F*_ST_ (for microsatellites) and *Φ*_ST_ (for mtDNA) values for the 10 populations were estimated using ARLEQUIN and their significance was tested using 1000 permutations corrected for multiple tests by Bonferroni correction. For comparative purposes, we have also calculated pairwise *D*_EST_ [41] using SMOGD v. 1.2.5 [42] (results shown only as Supporting information (S3 Table)). Isolation by distance (IBD) patterns were determined by comparing pairwise F_ST_/(1- F_ST_) [43] for both nuclear and mtDNA markers to the logarithm of geographical distance (measured in a straight line between the central point of each population except the distances to Norway and Finland which were taken along an arbitral broken line omitting the Baltic Sea) using Isolation by Distance Web Service (IBDWS) (http://ibdws.sdsu.edu/~ibdws/ [44].

Principal coordinate analysis (PCA) was performed based on a sample-wise matrix of genetic distance for microsatellite and mtDNA data in GenAlEx v. 6.0 [45]. We then used PC1 scores with the geographic coordinates of lynx samples for the spatial analysis of genetic differentiation, using the kriging algorithm in the Surfer 10 software (http://www.goldensoftware.com/demo-downloads). Contour lines of the first axis of the PCA were interpolated and superimposed onto a geographic map of the study area. By interpolating the PC1 scores for each individual lynx throughout the study area, we tried to identify regions where genetic dissimilarity between individuals was considerably higher or lower than would be expected from the IBD effect alone. Such geographic areas may represent dispersal barriers or migration corridors, respectively.

Analysis of molecular variance (AMOVA; [46] using ARLEQUIN (with 10000 permutations) was performed to assess structuring within the data, where the sampling sites were grouped as a single population (using both, *Φ*_ST_ and *F*_ST_). To explore patterns of genetic divergence in more detail, we applied the spatial AMOVA procedure using SAMOVA ver. 1.0 [47]. This allowed us to identify the grouping of sampling sites that maximized the *Φ_CT_* values (among group variance) and minimized *Φ*_SC_ values (among populations within group variance). The significance of *Φ*-statistics was tested using 10000 permutations for K = 2 to K = 9 partitions of the sampling sites. The p*R*_ST_ - *R*_ST_ tests between identified groups were performed in SPAGEDI [48], using 20000 allele size permutations to identify if genetic differentiation was due to genetic drift or other evolutionary processes.

Contemporary migration rates (within the past one to three generations) between lynx populations were evaluated using a Bayesian approach implemented in BayesAss ver. 1.3 [49] according to the authors' recommendations. The analyses were performed with 3×10^6^ iterations of which 10^6^ were burn-in and a sampling frequency of 2000. We further applied a Bayesian clustering approach to infer the most likely number of genetically distinct groups of samples using the software STRUCTURE 2.3.4. [50] without prior information of the sampling locations. We assumed the admixture model with correlated allele frequencies, and specified burn-in of 10^6^ iterations and 10^5^ Markov Chain Monte Carlo (MCMC) replicates after the burn-in period. The program was run 5 times for each number of clusters (K) between 1 and 10 to verify the consistency of estimates across runs. To determine most likely number of genetic clusters we evaluated the probability logarithm of the data (lnP(D); [50]) and Delta K (ΔK; [51]) for each K averaged across runs, using the software Structure Harvester (Web version: v0.6.93, [52].

We applied Approximate Bayesian computation (using the program DIYABC 2.0.4; [53]) to estimate the relative likelihood of possible scenarios for the lynx population histories. The program uses reference tables (containing parameters based on known or estimated values) to establish scenarios from which simulated data sets could be compared to the observed values. As the program requires a priori defining populations, for the analyses we generally followed the subdivision of the lynx population suggested by the STUCTURE analysis into four subpopulations: 1) Norway, 2) BPF-KARPF, 3) Baltic and 4) Russia-Finland. However, we decided to distinguish a fifth sub-population, the Carpathian population, due to the analysis in SAMOVA. We tested nine possible scenarios. We set the effective population sizes (Ne) from 10 to 10000. Each competing scenario was given equal prior probability. We accepted the default mutation rate model prior distributions suggested in the software for microsatellites. For the mitochondrial dataset we assumed the mean mutation rate of 5.35×10^−7^ substitutions/site/year with SD  = 2.28×10^−7^ substitutions [54]. We set the number of simulated data sets at 500000. We compared the relative likelihoods of the scenarios by the logistic regression approach, with a 2% subset of the closest simulated data. We provisionally assessed the fit of the model to the data using principal components analysis (PCA).

We tested for a possible sex-bias in dispersal using a spatial autocorrelation analysis [55] in GenAlEx 6.5 [45] for each sex separately. The autocorrelation coefficient, pairwise r values, were divided into 20 distance classes (100 km each) with mean sample sizes in each distance class N = 254 (±182). A null distribution of r values for each distance class was obtained by permutation (N = 9999) and the confidence intervals (C.I.) about r were estimated by bootstrapping with replacement (N = 9999), and plotted in a correlogram. The extent of the detectable spatial genetic structure was approximated as the distance class at which r was no longer significant and the intercept crossed the x-axis.

To identify correlations between genetic distance between lynx populations and abiotic climatic, as well as biotic factors (lynx basic prey), we performed multivariate multiple regression analysis using DISTLM ver. 5 [56]. The mtDNA and the microsatellite population pairwise *F*_ST_ values were used to construct the response matrix and tested against the following predictor matrices: geographical distance, average temperature in January, number of days with snow cover (DSC), average snow cover depth (SCD) in January [57], and the winter index for the North Atlantic Oscillation (NAO) as an index of climate [58]. Marginal tests of each predictor were done, followed by conditional tests, where latitude was included as a covariable to the predictor variables. Then, sequential tests were done using a forward selection procedure to produce a combined model of genetic differentiation in the Eurasian lynx using DISTLM forward ver. 1.3 [59]. The P values were obtained from 10000 permutations. Abiotic climatic data were taken from the WorldClimate database (www.worldclimate.com), [60] and the Climate Atlas of Poland [60]. Data obtained from the literature on lynx diet is cited in S4 Table.

## Results

### Intrapopulation genetic variability

**Figure 2:**
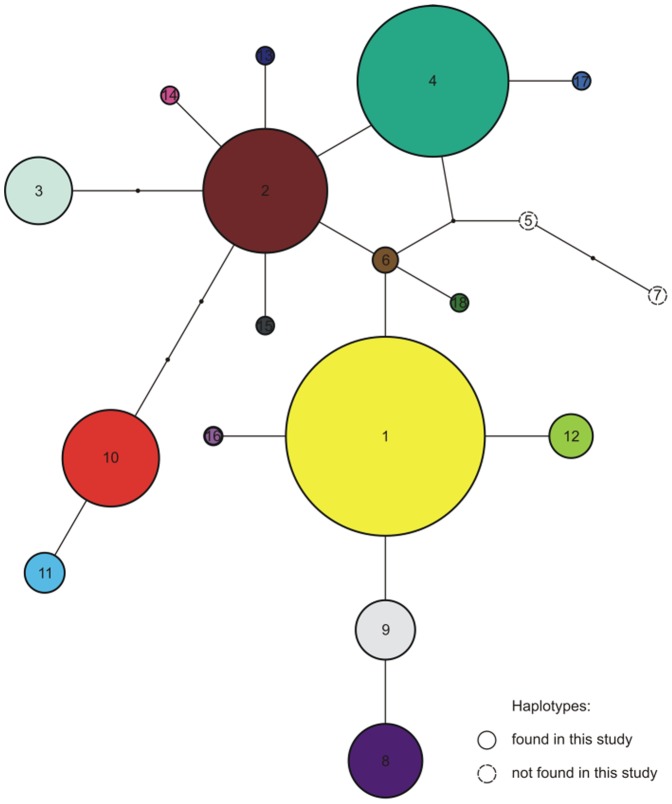
mtDNA haplotype network of Eurasian lynx. Haplotype network illustrating the relationship among 16 haplotypes of Eurasian lynx. Small black circles indicate missing haplotypes. Numbers denote the haplotypes. The size of the circles (except the haplotypes not found in this study) refers to the relative frequencies of a given haplotype in the whole sample. Colors of haplotypes correspond to Fig. 1.

We obtained control region mtDNA sequences for 332 lynx samples. Sixteen 613 bp haplotypes of the cr mtDNA were observed, of which four (H14, H15, H16 and H18) were previously unreported (Fig. 2). All new haplotypes are provided in GenBank (accession nos.: H14 - KM000076, H15 - KM000077, H16 - KM000078, H18 - KM000080). The number of segregating sites (S) was 18 (17 transitions and one indel). The number of haplotypes per population ranged from 1 (Norway and the Carpathians) to 8 (Russia; Table 1). Cr mtDNA H1 was the most widespread haplotype and it was present in eight of the sampled locations (Figs. 1 and 2). It was fixed in Norway and very common in Estonia, Finland and Russia. On the other hand, H4 was fixed in the Carpathians and relatively common (0.231) in Russia and was also present at moderate to low frequencies in Estonia, Latvia, Belarus and KARPF in Poland (Fig. 1, see S1 Table for haplotype frequencies). It is worth noting that three haplotypes were unique to Russia and, surprisingly, three were exclusively present in the Lithuanian samples. Haplotype (h) and nucleotide (π) diversity in the overall sample was 0.78 and 0.388%, respectively (Table 1). The haplotype diversity (h) values ranged from zero (Norway and the Carpathians) to 0.82 (Belarus). The Norwegian and Carpathian populations showed no nucleotide diversity (π = 0.000%), whereas this parameter was highest in Latvia (0.468%).

The cytochrome b gene (1094 bp) showed no polymorphism among 12 individuals from different populations (GenBank accession no.: KM000081). Likewise, no polymorphism was found among 681 bp of the entire *ATP6* gene among 8 lynxes from different populations (GenBank accession no.: KM000083).

No sequence polymorphism was found in the five Y-linked chromosome markers (total: 1386 bp; GenBank accession no.: KM000084, KM000085), among 43 male lynx from seven sampling locations (Russia, Finland, Estonia, Latvia, Poland, Romania and Belarus) and the Y-linked microsatellite locus 278g21-4 possessed only a single allele (146 bp long) in all males studied.

The number of alleles for the microsatellite loci ranged from four to 18 and we did not find any significant (Bonferroni-corrected) linkage disequilibria between loci within any of the populations studied, as well as the frequencies of null alleles low (below 0.05, data not shown). Measures of genetic diversity for the microsatellite loci (A, A_R_ and H*e*) were lowest for the Norwegian and BPF populations, and largest for lynx from Russia (Table 2). The A, A_R_ and H*e* values for Norway and BPF were significantly lower (permutation test, p<0.05) than for the remaining lynx populations, possibly due to bottlenecks. Interestingly, the Carpathian population did not show any signs of reduced levels of polymorphism at the microsatellite loci; however it exhibited a positive and significant *F*_IS_ value, as did the population from Belarus (Table 2).

**Table 2:** Microsatellite DNA diversity indices for the Eurasian lynx in Europe.

Sampling area	N	*N_A_*	*A_R_*	*He*	*F_IS_*
Norway	28	3.50	2.92	0.500	0.040
Finland	30	5.00	3.90	0.600	0.016
Estonia	61	5.33	3.69	0.590	0.002
Latvia	48	5.50	3.82	0.620	0.050
Lithuania	12	3.92	3.53	0.590	0.040
Belarus	11	3.75	3.60	0.600	0.163*
Poland (KARPF)	12	3.75	3.41	0.590	0.040
Poland (BPF)	22	3.50	2.85	0.477	0.012
Carpathians	13	4.33	3.80	0.600	0.163*
Russia (Kirov)	61	6.75	4.40	0.674	0.033
All	298	7.58	4.24	0.660	-

*N* – sample size, N_A_ – number of alleles, A_R_ – allelic richness, *He* – expected heterozygosity, *F_IS_* – inbreeding coefficient, * - p <0.05. Population abbreviations according to Table 1.

### Genetic differentiation among lynx sampling locations

Pairwise genetic differentiation values between sampling locations for the cr mtDNA ranged from 0.01 to 1.00 (*Φ*_ST_) and the majority of comparisons were significant (Table 3). These results indicate that a high degree of genetic differentiation exists between lynx populations, especially those from Norway, BPF and the Carpathians. Nonsignificant values of *Φ*_ST_ were found between Estonia and Latvia, Finland and Estonia as well as Latvia and KARPF, whereas lynx populations in close geographic proximity in Lithuania and Latvia showed considerable and significant mtDNA differentiation (Table 3). For the cr mtDNA *F*_ST_ was not significantly different from zero between three population pairs only: Estonia and Russia, Latvia and Belarus, Belarus and KARPF (S2 Table). Similarly, various degrees of genetic differentiation were found at the microsatellite loci, with pairwise *F*_ST_ values ranging from 0.012 to 0.293 and the highest divergence between the populations of Norway, BPF and Carpathians with the other sampling locations (Table 3). All comparisons except one (the Latvia-Lithuania population pair) were significant. Comparative patterns of differentiation were obtained with *D*_EST_ which ranged from 0.00 to 0.32 (S3 Table). The *R*_ST_ values (S3 Table) ranged from 0.004 to 0.234 and the majority of comparisons were significantly different from zero. The *R*_ST_ values were non-significant between the following population pairs: Finland - Lithuania, Estonia - KARPF, Estonia - Lithuania, Latvia - Lithuania, Lithuania - Belarus, Lithuania - KARPF, Lithuania - Carpathians, Lithuania - Russia, KARPF - BPF, KARPF - Russia.

**Table 3:** Pairwise differentiation between Eurasian lynx populations.

Population	Norway	Finland	Estonia	Latvia	Lithuania	Belarus	KARPF	BPF	Carpathians	Kirov
Norway	-	0.23	0.13	0.36	0.94	0.57	0.68	0.92	1	0.19
Finland	0.187	-	*0.06*	0.24	0.67	0.32	0.51	0.82	0.87	0.09
Estonia	0.153	0.059	-	*0.09*	0.37	0.14	0.33	0.67	0.59	*0.02*
Latvia	0.158	0.041	0.007	-	0.14	*0.01*	*0.12*	0.59	0.45	0.10
Lithuania	0.189	0.035	0.041	*0.012*	-	0.19	0.13	0.78	0.90	0.43
Belarus	0.185	0.076	0.081	0.082	0.09	-	*0.03*	0.56	0.69	0.15
KARPF	0.201	0.081	0.102	0.071	0.052	0.125	-	0.48	0.60	0.36
BPF	0.293	0.159	0.189	0.149	0.123	0.225	0.029	-	0.92	0.72
Carpathians	0.242	0.143	0.157	0.142	0.144	0.166	0.188	0.257	-	0.64
Kirov	0.146	0.038	0.048	0.043	0.052	0.060	0.078	0.149	0.121	-

*F*_ST_ values based on 12 microsatellites (below diagonal) and *Φ*_ST_ values based on the mtDNA control region (above diagonal). Nonsignificant values are given in italics.

**Figure 3:**
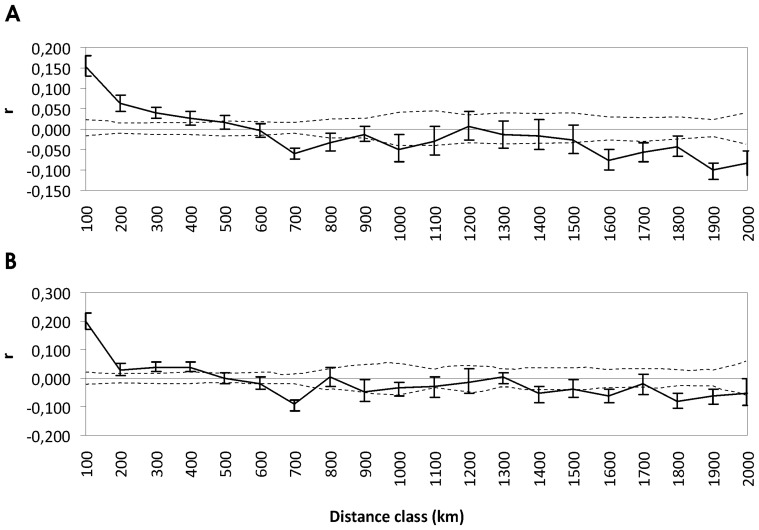
Results of the spatial autocorrelation analysis. Correlograms of the average autocorrelation coefficient (r) for 20 distance classes of 100 km each for male (A) and female (B) Eurasian lynx. The dashed lines represent the 95% upper and lower bounds of the null distribution assuming no spatial structure. The error bars represent the 95% confidence intervals about r. Significant spatial structure is observed when r exceeds the null distribution and the error bars do not overlap zero.

Spatial autocorrelation among females and males showed a pattern of decreasing relatedness with increasing distance in the first two distance classes (0–200 km), with an x-intercept at 498 km and 586 km for females and males, respectively (Fig. 3). For females, the r values were positive and significant for the first four classes (up to 400 km), while for males the corresponding values were significant up to 500 km. Thus, the approximate scale of the positive genetic structure was between 400 and 500 km. The overall shape of the correlogram was similar for males and females (Fig. 3), though with slight indication of male-bias in dispersal distance. The relationship between r and distance was not significantly negative until 700 km for females and males (Fig. 3).

We did not find any statistically significant isolation by distance (IBD) pattern for mtDNA (r^2^ = 0.028, p = 0.28) nor for microsatellite loci (r^2^ = 0.076, p = 0.17) among our sampling locations (S1 Fig.). When we excluded lynx from Russia, the IBD pattern became significant for microsatellite loci (r^2^ = 0.394, p<0.05) but not for the cr mtDNA (r^2^ = 0.178, p = 0.08) (S1 Fig.).

Geographical structuring among lynx cr mtDNA haplotypes in the study area was highly supported by AMOVA results where all sampling sites were treated as a single group (*Φ*_ST_ = 0.570, p<0.001, *F*_ST_ = 0.464, p<0.001). SAMOVA was subsequently used to identify the subdivision that most likely explains the mtDNA structure observed in lynx. The data were best explained assuming four groups of lynx populations: (1) Norway, (2) BPF, (3) Carpathians and (4) the remaining 7 sampling sites (Fig. 1). This configuration maximized the among-group variation (44.24%) and minimized the variation among sampling locations within groups (13.67%); the variation within sampling locations was 42.10% (*Φ*_CT_ = 0.442, p<0.05, *Φ*_SC_ = 0.245, p<0.001, *Φ*_ST_ = 0.579, p<0.001).

For the microsatellite data, AMOVA supported the geographical structuring of the populations (*F*_ST_ = 0.10, p<0.001, *R*_ST_ = 0.09, p<0.001). The data were best explained in SAMOVA analysis assuming the same four groups of lynx populations: (1) Norway, (2) BPF, (3) Carpathians and (4) the remaining 7 sampling locations (Fig. 1). This configuration maximized the among-group variation (11.81%) and minimized the variation among sampling locations within groups (4.38%); the variation within sampling locations was 83.81% (*F*_CT_ = 0.118, p<0.01, *F*_SC_ = 0.050, p<0.001, *F*_ST_ = 0.162, p<0.001). *R*_ST_ did not differ significantly from p*R*_ST_ for any pairwise between-group comparisons (P>0.27, 1000 permutation tests).

**Figure 4:**
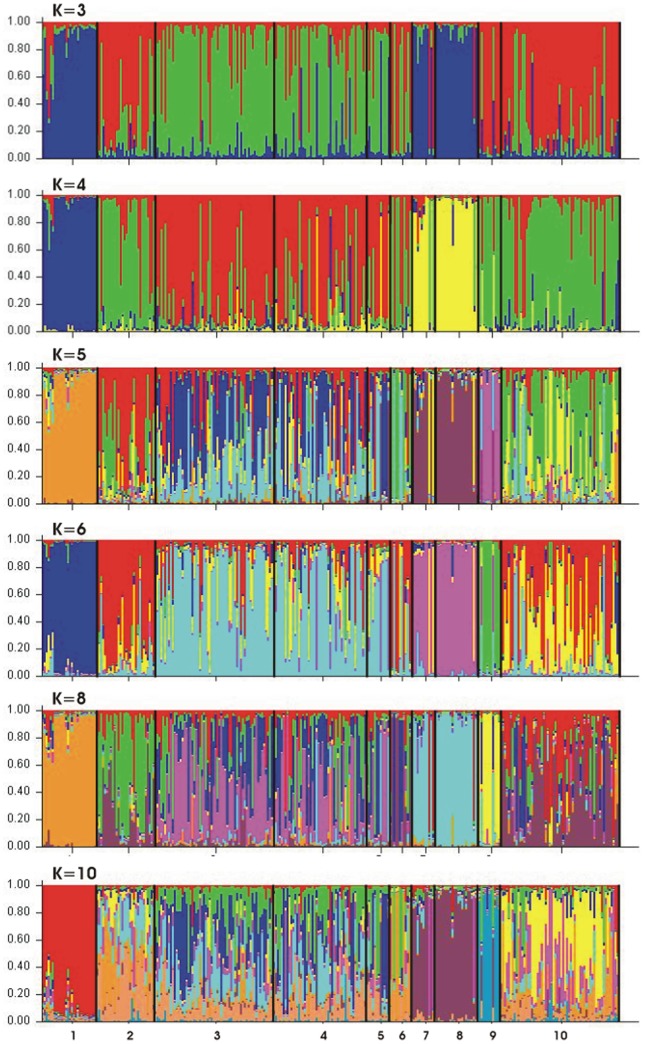
Bayesian clustering of the Eurasian lynx population in north-eastern Europe. Results of the STRUCTURE analysis assuming number of clusters from K = 3 to K = 10. Numbers correspond to classification of populations in Table 1. Note consistency of populations: 1 (Norway), 7 and 8 (KARPF and BPF, both NE Poland) being composed of two uniform genetic clusters across all values of K.

Using the Bayesian clustering method based on multilocus lynx genotypes with STRUCTURE indicated that four genetic clusters were most likely using the ΔK method (S2 Fig.). However, with the probability logarithm of the data (lnP(D) method, although the likelihood values leveled off at K = 4, it reached a maximum at K = 8 suggesting further subdivision of the lynx population (S2 Fig.). About 75% of the individuals were assigned to genetic clusters using Q>0.80 as a threshold, with considerable genetic mixing between two of the different clusters. The genetic assignment plots produced in STRUCTURE clearly highlighted the genetic discontinuity among lynx populations. Assuming K = 4, the samples were assigned according to the following pattern: two sampling locations (the Norway and the North-Eastern Polish lynx, including the BPF and KARPF) were nearly uniformly composed of two different single genetic clusters, a third cluster was composed of lynx from Finland, Russia, Carpathians and to some extent from Belarus, whereas the fourth cluster included the remaining lynx (Estonia, Latvia, Lithuania and Belarus) (Fig. 4). Interestingly, irrespective of the number of clusters assumed, the Norwegian and the North-Eastern Polish lynx populations always formed clearly distinguishable genetic units.

### Contemporary gene flow and possible scenarios of past lynx population history

**Figure 5:**
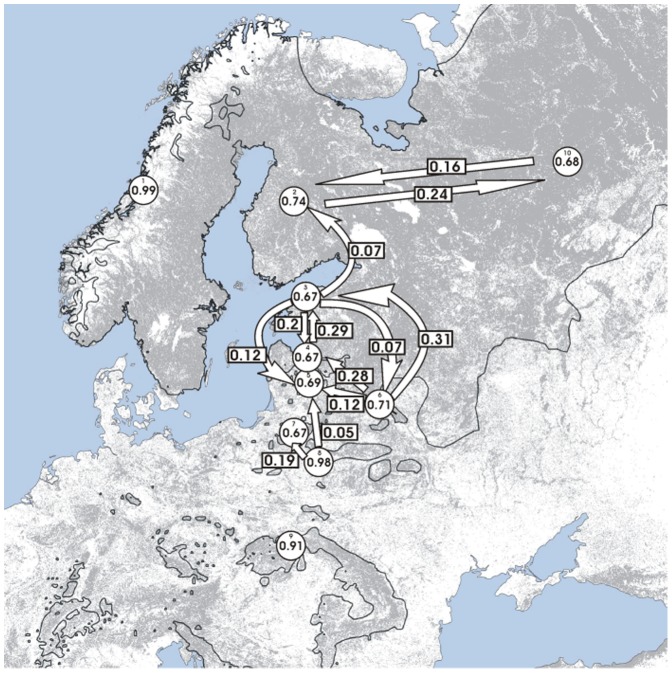
Migration routes and rates of Eurasian lynx across north-eastern Europe. Recent migration rates within the Eurasian lynx population in north-eastern and central Europe between arbitrarily assigned sampling populations, estimated using BayesAss. Directions and rates of migrations are shown with arrows and associated numbers. The numbers within the circles denote proportions of non-immigrants within the sampled populations (denoted with small digits which refer to the population numbers and names in Table 1). Grey shading represents the forest cover. It is prepared based on an open access GlobCOVER database (http://due.esrin.esa.int/globcover/) by extracting a range of data indicating forested and non forested areas (limited to values of 40–110). It is thus similar but not identical to the original image, and is therefore for representative purposes only. The lynx range (after Von Arx *et al*. [91]) is marked by continuous lines.

Bayesian estimates of contemporary migration estimated in BayesAss were close to zero for Norway, BPF and the Carpathians. Some migration probably occurred from BPF into the KARPF population and to a certain extent from BPF to Lithuania. The lynx population from Belarus could act as a source population for the neighboring populations in the Baltic states. Regular recent migration was evident between Finland and Russia in both directions (Fig. 5).

**Figure 6:**
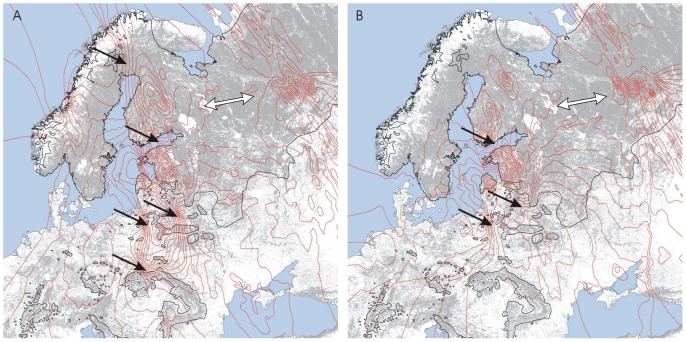
Results of spatial analysis of genetic differentiation in the Eurasian lynx. Maps showing contour lines of the first axis of the PCA performed on a sample-wise matrix of genetic distance for microsatellite (A) and mtDNA (B) data interpolated with the use of the kriging algorithm (Surfer12 software) and superimposed onto a geographic map of the study area. Black and white arrows show possible migration barriers or migratory corridors respectively. See Fig. 5 for other explanations.

The synthetic genetic map based on PC1 scores calculated from pairwise genetic distance between individuals, allowed us to identify potential dispersal barriers and migration corridors (Fig. 6). Microsatellite data set revealed areas of abrupt genetic change, indicating that possible barriers to lynx dispersal may exist in: (1) the northern part of the Scandinavian peninsula, (2) the gulf of Finland, (3) the highly fragmented habitats around NE Poland and (4) the deforested area north to the Carpathians (Fig. 6A). On the other hand, fairly homogeneous genetic landscape was visible between southern Finland, eastern Belarus and western Russia, suggesting a possible migration corridor in this part of the study area (Fig. 6A). The analogous migration corridor was also revealed based on the mtDNA data set, but in contrast to microsatellites, no barriers were detected in northern Scandinavia and north of the Carpathians (Fig. 6B). Relatively high genetic dissimilarity between sampled lynxes was visible on the mtDNA contour map near the gulf of Finland and in NE Poland (Fig. 6B).

**Figure 7:**
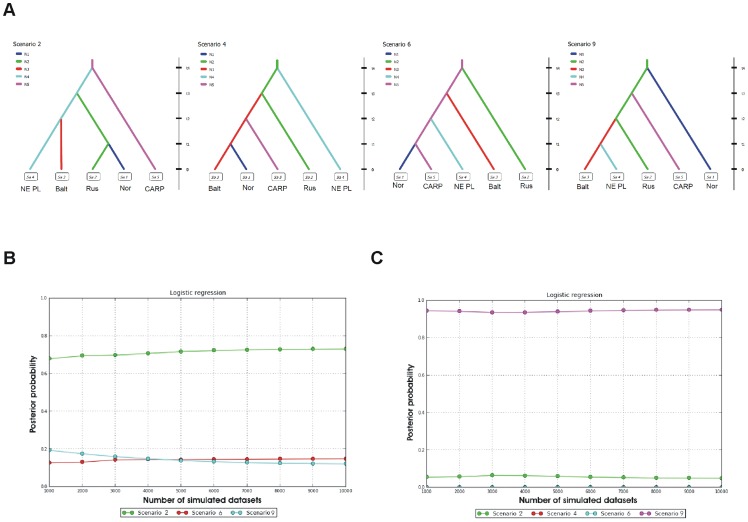
Possible scenarios of Eurasian lynx population history. Results of the approximate Bayesian computation conducted with the use of DIYABC 2.0.4 [53] to estimate the relative likelihood of alternative scenarios for the lynx's colonization history. A) Graphs in the upper panel illustrate the four final best supported scenarios from among nine scenarios proposed in the analyses (see S2 Appendix for details). The ten sampling locations analyzed in this study were grouped into five populations based on its distinctness suggested by the STRUCTURE and SAMOVA analyses: 1) Nor (Norway), 2) NE PL (BPF and KARPF), 3) Balt (Latvia, Lithuania, Estonia, Belarus), 4) Rus (Russia and Finland) and 5) CARP (Carpathians). Colors in the colonization scenarios indicate different (but unknown) population sizes (Ne). Zero means sampling time and t1-t4 mean relative times of past events of suggested population splitting. Graphs in the lower panel indicate the relative likelihoods of the four best scenarios compared by a logistic regression based on microsatellite (B) and mtDNA (C) data with a 2% subset of the closest simulated data. The best support is for the scenario 2 and 9 for microsatellite (B) and mtDNA (C) data, respectively. Note, although four scenarios were analyzed for mtDNA (C) the posterior probability of scenario 4 is not visible due to highly similar values with scenario 6.

Using the approximate Bayesian computation with DIYABC we found highest support for two different (but very consistent through various approaches; see S2 Appendix), mutually exclusive scenarios of lynx population history depending on the genetic marker used. The analysis based on microsatellites suggested that the lynx from the Carpathian mountains could have been the source for all remaining populations (Fig. 7, Scenario 2). The first split, according to this scenario, has lead to the establishment of the North-Eastern Polish population of lynx (BPF and KARPF). During the second split a branch containing the Russian, Norwegian and Finnish lynx split off from the NE Polish branch. The latter population diverged also at a subsequent split giving rise to the Baltic population. During the last, most recent split, the Norwegian lynx separated from the Russian-Finnish branch.

The mtDNA-based DIYABC analysis, however, showed that it is the Russian-Finnish population that was the source population from which the Norwegian lynx has split first (Fig. 7, Scenario 9). The Russian-Finnish lynx gave also rise to the Carpathian and Baltic populations at the second and third splits, respectively. During the most recent split the Baltic population has given rise to the NE Polish lynx.

### Possible effect of ecological factors on lynx genetic structure

A test of the influence of geographical location on genetic differentiation among the lynx sampling locations showed that pairwise *Φ*_ST_ values (mtDNA) is correlated with latitude (46.5% of variation explained, P<0.001) but not with longitude. Similarly, the number of days with snow cover, snow cover depth, and the NAO index, as revealed by marginal tests, had significant effect on mtDNA differentiation among sampling locations (Table 4). When geographical coordinates were incorporated as covariables into the multiple regression analysis (conditional tests), only snow cover depth was correlated with the pairwise *Φ*_ST_ values between the lynx sampling locations. On the other hand, sequential tests showed that latitude and snow cover depth were the only significant factors (Table 4). The effect of snow (SCD and DSC taken as a set of variables) explained 59.3% of the variation (p<0.01) for the mtDNA data in marginal tests. No such relationship was found for the microsatellite data except in one analysis. There was a significant effect of the main prey and the share of red deer in the lynx diet explaining 82.5% (p<0.05; the same for sequential tests) and 26.6% (p<0.05) of the variation, respectively, in the marginal tests for microsatellites only (Table 4).

**Table 4:** Effects of abiotic climatic variables on genetic differentiation based on mtDNA and microsatellites among Eurasian lynx populations in Europe.

Environmental factor	Marginal tests		Conditional tests		Sequential tests	
	% var	*P*	% var	*P*	% var	*P*
Latitude	0.465	<0.001	n.a.	-	0.465	<0.001
	*0.271*	*0.058*	*n.a.*	-	*0.142*	*0.118*
Snow cover depth	0.358	0.001	0.260	<0.05	0.260	<0.05
	*0.193*	*0.158*	*0.114*	*0.294*	*0.157*	*<0.05*
Longitude	0.001	0.990	n.a.	-	0.096	0.093
	*0.020*	0.*734*	*n.a.*	-	*0.080*	*0.080*
Days with snow cover	0.389	<0.01	0.135	0.243	0.029	0.190
	*0.212*	*0.116*	*0.161*	*0.483*	*<0.001*	-
Snow	0.593	<0.01	0.295	0.097	0.193	0.264
	*0.311*	*0.212*	*0.483*	*0.161*	*0.110*	*0.291*
NAO	0.425	0.001	0.177	0.112	0.020	0.690
	*0.237*	*0.080*	*0.068*	*0.522*	*0.043*	*0.133*
Temperature in January	0.121	0.389	0.033	0.511	<0.001	-
	*0.041*	*0.724*	*0.091*	*0.417*	*0.035*	*0.999*
Prey	0.709	0.207	0.393	0.491	0.709	0.207
	*0.825*	*<0.05*	*0.553*	*0.430*	*0.825*	*<0.05*
Red deer	0.212	0.161	0.094	0.357	0.104	0.181
	*0.266*	*<0.05*	*0.215*	*0.114*	*0.187*	*0.072*

Marginal, conditional and sequential tests of the forward selection procedure are reported. Percentage of genetic variation explained by a particular variable (% var), probability values (*P*), upper line - mtDNA data, bottom line (*in italics*) - microsatellites. NAO – North Atlantic Oscillation Index; Snow – snow Cover Depth (SCD) and Days with Snow Cover (DSC) together. Prey – the proportion of main prey species in the lynx's diet (see Appendix 1). n.a. – not applicable since coordinates were used as covariables in conditional tests, (-) – not tested.

## Discussion

### Population differentiation

The important finding of our research is the presence of similar patterns of genetic differentiation among populations based on two types of markers: mtDNA and microsatellites in the Eurasian lynx. The entire sample was consistently subdivided into four genetic units. SAMOVA also suggested that while lynx from a large area encompassing Finland, the Baltic countries, Belarus and Russia were all included in a single genetic unit, the three remaining sampling locations – Norway, the Carpathians and the Białowieża Primeval Forest (BPF) were separated into three clearly distinct genetic entities. Based on the different heritability of mtDNA and microsatellites, as well as previous research that suggested different genetic structure in the brown bear with use of the same types of markers [21], [22], we expected to find less consistency when considering both mtDNA and microsatellite variability in the Eurasian lynx.

Spatial autocorrelation analysis provided relatively little evidence for male-biased dispersal in the lynx. This is not fully concordant with the available telemetry data, suggesting that similar to other large felids [61]–[63], male lynx disperse more frequently and for longer distances than females [64]–[65]. This may mean lower effective dispersal in this species than expected from observational data. The existence of microsatellite genetic structure almost as strong as that found with mtDNA (which is inherited solely through females) indicates that there may be effective physical barriers to male dispersal and that female philopatry alone does not explain the observed pattern. Such barriers are likely to function in the form of habitat fragmentation. It is particularly visible on the synthetic genetic map in the case of the BPF population, which showed a remarkable genetic distinctness from the neighboring populations despite the short geographic distance between them. Indeed, the habitat seems most severely fragmented in this part of the lynx range [12], [66], and its effect was already reported in an earlier study based on six microsatellites [31]. Concordant genetic structure in the two types of markers was also recently found by Czarnomska *et al*. [14] in the wolf population inhabiting Poland and was explained by factors including natal-habitat biased dispersal and habitat disparities. The effect of habitat discontinuity on dispersal and therefore the population genetic structure in lynx may be more direct due to their stronger dependence on forest habitat [67], [68] as compared to that of wolves [69]. It is thus likely that effective dispersal may not be sufficient to counteract the effect of genetic drift in local, isolated populations of the Eurasian lynx. Indeed, no significant differences between *R*_ST_ and p*R*_ST_ values for microsatellite loci suggest genetic drift as the primary reason for observed genetic divergence among the lynx populations at microsatellite nuclear loci.

The strong genetic structure in the Eurasian lynx revealed by our analyses corresponds well with our previous study on mtDNA that showed large differentiation among the populations at the westernmost peripheries of its range [26]. However, sampling more locations in this study, particularly from the core of lynx range (Russia, Kirov), and applying microsatellite markers gave us more explanatory power with regards to plausible factors influencing the observed pattern of genetic variability in this carnivore. The isolation by distance (IBD) pattern appeared significant only for microsatellites for the data set without the Russian population. This suggests that the population differentiation at the westernmost part of the species' range may be dependent on the dispersal capabilities of lynx and IBD may thus explain only a portion of the observed pattern of population differentiation. With the inclusion of the Russian subsample the IBD was no longer significant. This suggests that there must be no particular genetic divergence between the core (Russia) and some peripheral parts (e.g. Finland, Belarus or Estonia) of lynx's range. Thus, the Eurasian lynx has the potential to maintain a panmictic population encompassing the vast territory of Eastern Europe, unless it encounters barriers due to habitat fragmentation. The spatial, individual-based, analysis of genetic distance showed that it is plausible that deforested areas at the western margins of the Eurasian lynx range act as effective barriers to dispersal between the BPF, Carpathians and the other studied populations. The high genetic gradients on the synthetic genetic maps along with low genetic diversity (for the microsatellites) of the Norwegian and the BPF samples suggests that they have both experienced genetic drift and are particularly restricted by barriers to dispersal. The barrier between northern Norway and Finland is most probably caused by intense hunting pressure in areas where reindeer are present [70].

Our study has also indicated that the genetic structure in the Eurasian lynx may be explained, at least in part, with climatic conditions, particularly the depth of snow cover, which was significantly correlated with mtDNA population differentiation, even when geographic coordinates were included as co-variables in the conditional tests. The lack of a barrier on the synthetic genetic map for mtDNA in northern Scandinavia throughout Finland and Russia coincides well with similar climatic conditions (e.g. high snow cover depth) in all northern lynx populations. Very similar results have recently been reported based on mtDNA for a smaller dataset of lynx samples from the western part of its range [12]. The mechanism suggested for this relationship involves the matrilineal inheritance of mtDNA as well as the fact that the climatic factors may also have affected the kill rate by female lynx [71]. Moreover, skull size in female lynx, but not males, was also found to be influenced by climatic factors [72]. Females in solitary cats are provisioning their offspring with food alone [73], so familiarity with local snow conditions may indeed influence their hunting efficiency and in effect the survival and development of their kittens.

The relationships between microsatellite population differentiation and climatic factors were less clear, with the only significant effect of snow conditions found in the sequential test. On the other hand, a significant effect on microsatellite differentiation in the marginal and sequential tests were found for prey, especially the proportion of red deer in the lynx's diet. Although drawing firm conclusions from the latter result can be somewhat speculative, red deer are killed more often by lynx in North-Eastern Poland (BPF and KARPF) than in other areas (S4 Table and citations therein), which coincides with the genetic distinctness of the lynx inhabiting this region.

The results of multivariate multiple regression, along with the IBD effect may suggest that despite generally concordant patterns of population subdivision, there might be different factors such as climate and prey type affecting mtDNA and the microsatellites genetic structure of lynx population, respectively. The effect of climate may have resulted in the matrilineal clusters of lynx being specifically adapted to hunting prey under local snow conditions. Moreover, as male lynx are more likely to kill larger prey [74], [75] one cannot exclude the possibility of lynx microsatellite genetic structure being additionally affected by male adaptations to killing red deer. The influence of prey specialization on predator population structure has already been well documented in the wolf [10], [11], and inferred for the Canada lynx (*Lynx canadensis*) [8].

### Gene flow among lynx populations

This study revealed differing levels of gene flow within the range of the Eurasian lynx. Low genetic divergence and high rate of migrants identified between the eastern part of European Russia and some western lynx sampling locations suggested high gene flow along the east-westward direction, despite the distance between locations. Several independent analyses of our microsatellite data indicated possible migration corridors situated between Russia (Kirov and Arkhangelsk Oblast), Finland and Belarus. In contrast, little genetic exchange appears to have occurred between neighboring populations situated at the westernmost peripheries of the lynx range, e.g. Norway, the BPF and the Carpathians.

The possibility of recent gene flow between lynx inhabiting the far east of European Russia and the westernmost edge of the lynx range (approximately 1100–1300 km) may not be surprising considering the long distances large carnivores (including lynx) are capable of covering during dispersal and migrations [2], [63], [65], [76]. Nevertheless, our findings indicated, for the first time, the effectiveness of this mobility in a genetic sense. Previous genetic studies conducted at smaller scales were mainly focused on documenting how gene flow was limited in this carnivore, despite the high dispersal potential [24], [25], [26], [31].

Studies documenting long-distance, cross-continental movements in wildlife are rare due to difficulties in collecting representative material [2], [10], [15], [16]. The existence of a migration corridor in Eurasia was revealed recently for brown bear between Eastern European Russia and Finland by Keis *et al.* [15] and the authors suggested that the corridor they detected coincided with the southern border of the taiga biome. High gene flow across a large area of Eastern Europe was also found in the grey wolf [10], [16]. The feasibility of gene flow documented in large carnivores corresponds with the distribution of continuous forest cover, which spans Eastern Europe from Latvia, Estonia and Finland to Eastern Russia [66] and the habitat preferences of all three of these large carnivores [67], [68], [77].

The lynx population inhabiting eastern European Russia was characterized by high genetic variation, and therefore one may expect it to constitute a possible source population for these cats in Europe. However, we found present-day gene flow between Russian and Finnish lynx in both directions with migration rates slightly higher from Finland to Russia than in the opposite direction. The pattern of lynx population history revealed with DIYABC analysis of the mtDNA data supports the scenario of a Russian origin for the European lynx populations.

### Genetic variability and contemporary and historical bottlenecks

The highest variability at both types of markers was found in sampling locations grouped into clusters situated within the core lynx range (Russia) or being directly connected to it through the continuity of the habitat present (Latvia, Estonia). Whereas high genetic variability in this cluster can be directly linked with the central location and habitat continuity [78], the reasons for the very low variability in each of the remaining three clusters (consisting of BPF, Norway and Carpathians) may have a unique reason in each case.

Among the three most divergent and least variable populations, the Norwegian and Carpathian lynx present different patterns of genetic variability. Whereas the lynx in both areas showed a complete lack of polymorphism in mtDNA, they had different levels of microsatellite variability. As the Norwegian lynx had one of the lowest reported values for allelic richness and heterozygosity, it is clearly concordant with the relatively recent bottleneck experienced by this population during the twentieth century [24], [79]. In contrast, the Carpathian lynx showed relatively high genetic variability in microsatellites, which indicates that no dramatic reduction of population numbers occurred recently. In this case the presence of only one haplotype of cr mtDNA may support the northern refugium hypothesis for the lynx with small effective population size during the last glacial maximum (LGM, [26], [80], [81]. This scenario is also in line with the statistical analyses in which the SAMOVA indicated that the Carpathian lynx formed a separate genetic group for both mtDNA and microsatellites, and the DIYABC analysis of microsatellite data, which suggested the Carpathian population to be the source for all the other lynx populations. The best supported scenario in this study, which implied the spread of the lynx from Carpathian mountains north and eastwards was also recently suggested by Rueness et al. [25]. The Bayesian approach in STRUCTURE suggested the Carpathian population to be composed of a single genetic cluster along with the Russian lynx. The haplotype H4 was found to be common in other sampling locations and frequent in the core population (Russia). Thus our results suggest that the present monomorphism in cr mtDNA in the Carpathian lynx could have been attributed to the bottleneck effect during the LGM. It is likely that the lynx population became extinct north of the Carpathians, and the few survivors reached these mountains and quickly established the population there. This might have deterred possible female migrants from settling in the existing social structure of the population [12] after the glacier retreated and resulted in the fixation of the single haplotype.

In the case of the BPF population, it is most likely that both its peripheral location and strong isolation from the core range were the main reasons for the low microsatellite and mitochondrial variability. A similar relationship between genetic variability and location relative to the core of the species range was reported for the Canada lynx [78] and the effect was explained with small population size and limited connections to other populations, which is typical in peripheries of a species' range [82]. The limited genetic variability observed in the BPF population might also be related to the short-term bottlenecks that most probably occurred during 19th and 20th centuries [83], causing genetic drift.

## Conclusion

The Eurasian lynx is capable of maintaining a panmictic population across eastern Europe unless they are severely limited by habitat continuity or a reduction in numbers. The effect of climatic conditions (snow cover) on the genetic divergence between populations with respect to mtDNA, but not nuclear microsatellite loci, may suggest different selective pressures acting on males and females in solitary carnivores. However, further research is needed to identify such selective pressures.

## Supporting Information

S1 Fig **Results of isolation by distance (IBD) analysis of the Eurasian lynx populations.** Two upper panels show IBD for microsatellite data for all ten putative populations (A) and excluding Kirov (Russia) (B). Two lower panels show IBD for mtDNA data for all populations (C) and excluding Kirov (Russia) (D). A significant relationship was only obtained for microsatellite data after excluding the Russian population.
(PDF)

S2 Fig **Number of genetic clusters of the Eurasian lynx population in north-eastern Europe.** Evaluation of the most likely number of genetic clusters with the ΔK method (A) and the probability logarithm of the data (lnP(D) (B). ΔK clearly suggests that K = 4 is the most likely number of clusters, while lnP(D|K) shows leveling off from K = 4 and the highest likelihood at K = 8.
(PDF)

S1 Table **Haplotype frequencies of the mtDNA control region in ten populations of Eurasian lynx** ***Lynx lynx*** **.**
(DOC)

S2 Table **Pairwise differentiation between Eurasian lynx populations.** *F*_ST_ values based on 12 microsatellites (above diagonal) and *F*_ST_ values based on the mtDNA control region (below diagonal). Nonsignificant values are given in italics.
(DOC)

S3 Table **Pairwise differentiation between Eurasian lynx populations.** *D*_EST_ (above diagonal) and *R*_ST_ (below diagonal) values based on 12 microsatellites.
(DOC)

S4 Table **Habitat type, climate and lynx diet composition of the ten lynx populations studied.** * References used for data on lynx diet: Norway [84]; Finland: [85]; Estonia and Latvia: [86]; Belarus: [87]; Poland (KARPF): [88]; Poland (BPF): [27]; Carpathians: [89]; Russia (Kirov): [90]. No data were available for Lithuania – for the computations we arbitrarily assumed the same prey composition as in Latvia due to very close geographical proximity. The data were recalculated from original sources considering the relative share of only key prey that occurred in all populations.
(DOC)

S1 Appendix **List of Eurasian lynx genotypes based on 12 microsatellite loci.**
(XLS)

S2 Appendix **Procedures adopted in the Approximate Bayesian Computations (DIYABC 2.0.4 software) and the list of all scenarios tested.**
(DOC)
