# Supplementary figures and images for: Long-Range Gene Flow and the Effects of Climatic and Ecological Factors on Genetic Structuring in a Large, Solitary Carnivore: The Eurasian Lynx
Source: PLoS One. 2014 Dec 31;9(12):e115160. doi: 10.1371/journal.pone.0115160 (PMC4281111; doi:10.1371/journal.pone.0115160)

A

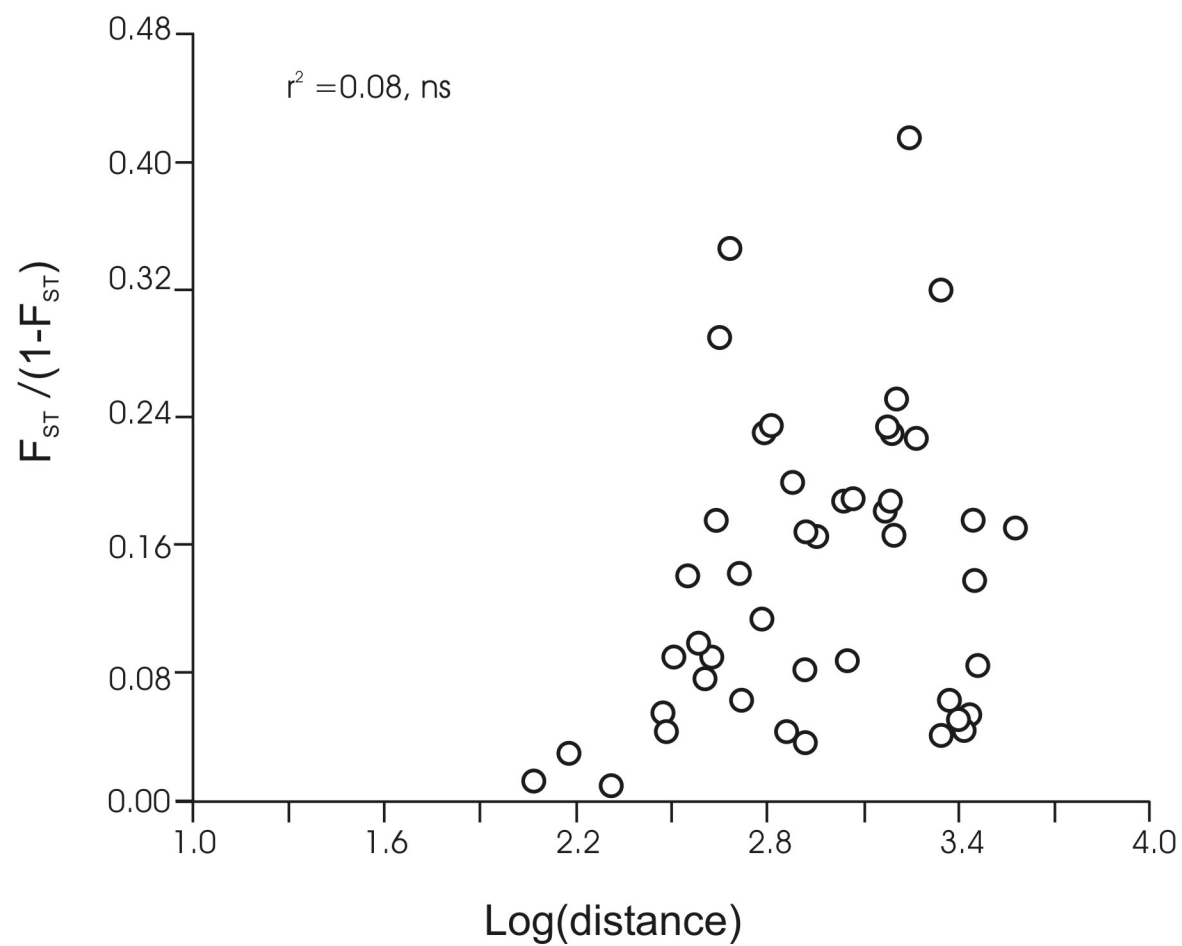

B

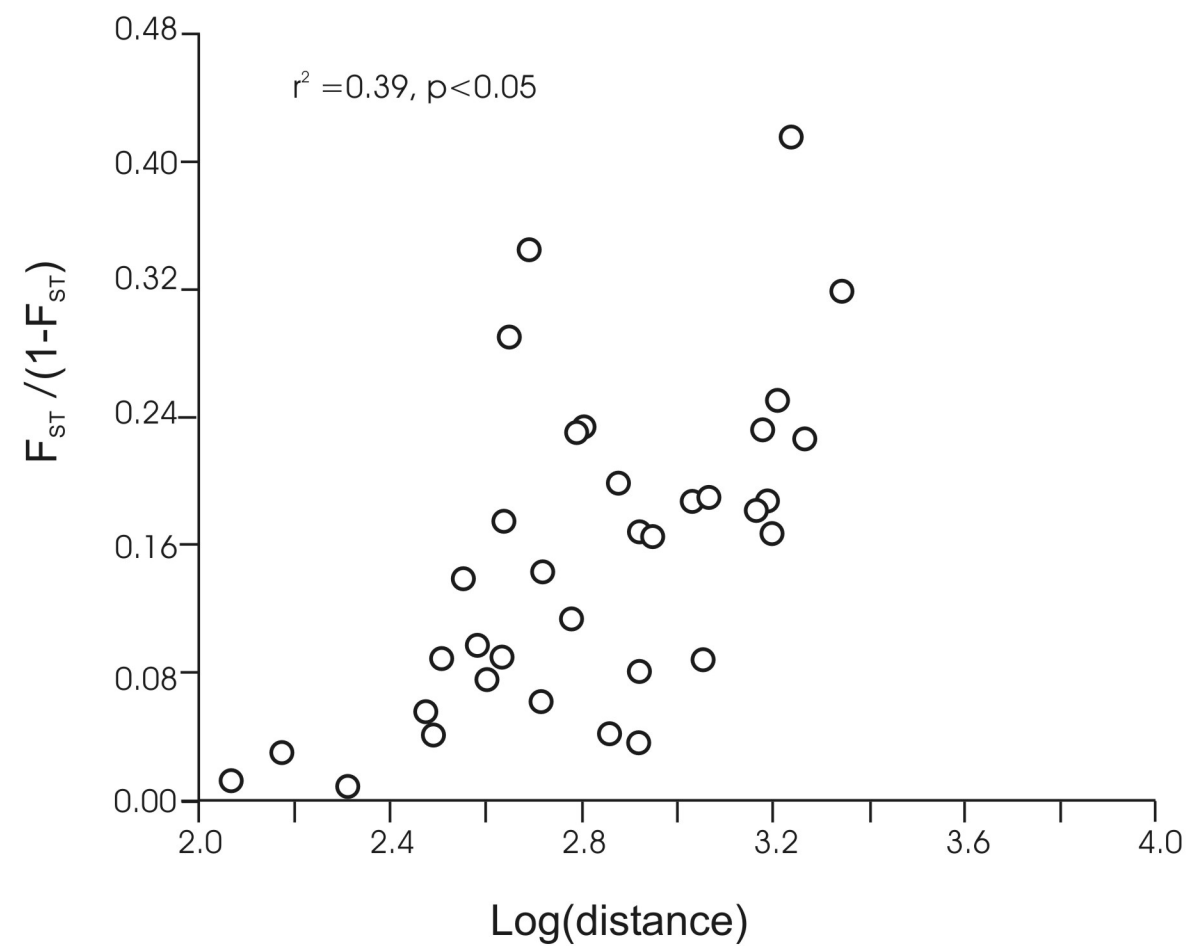

C

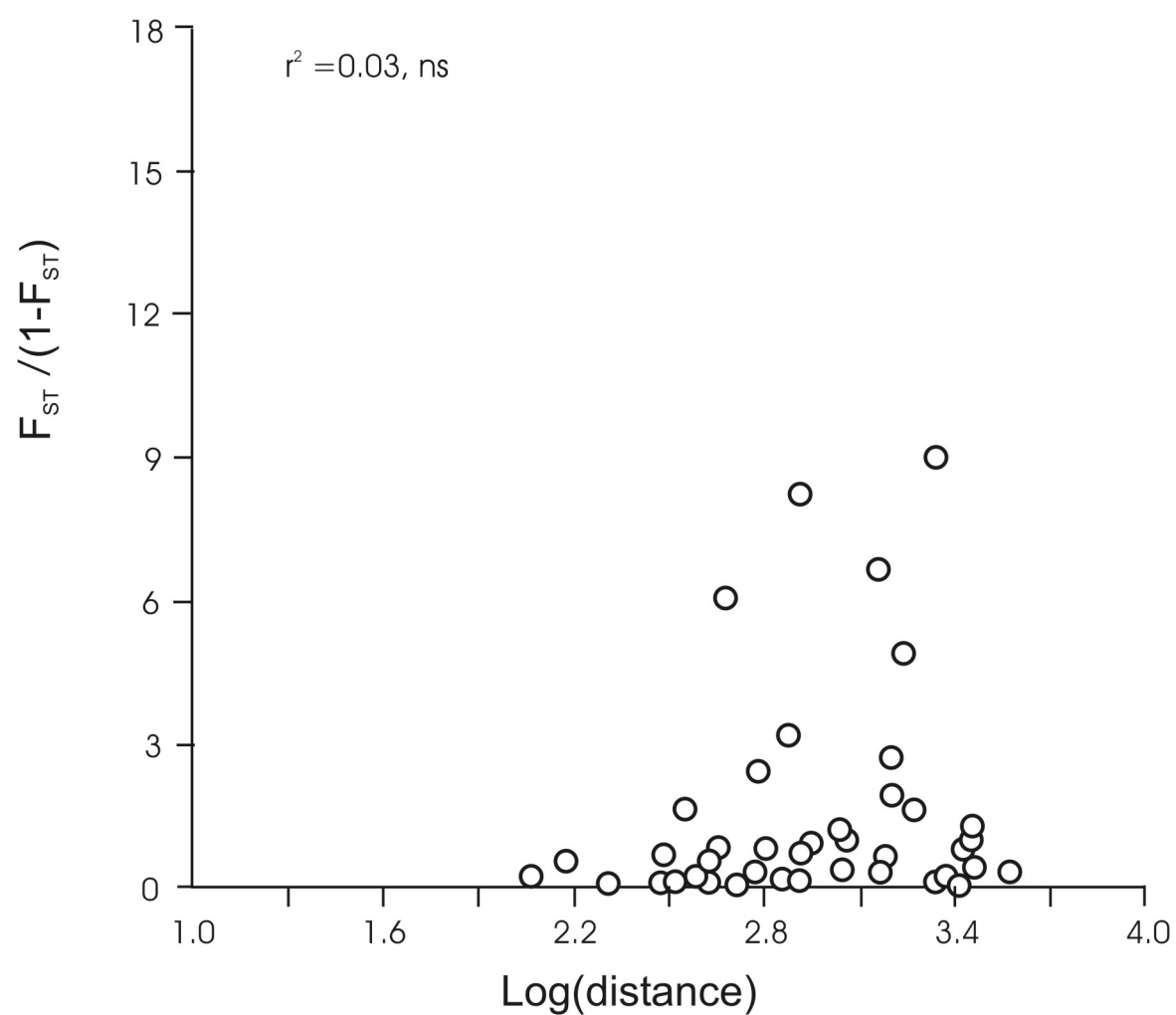

D

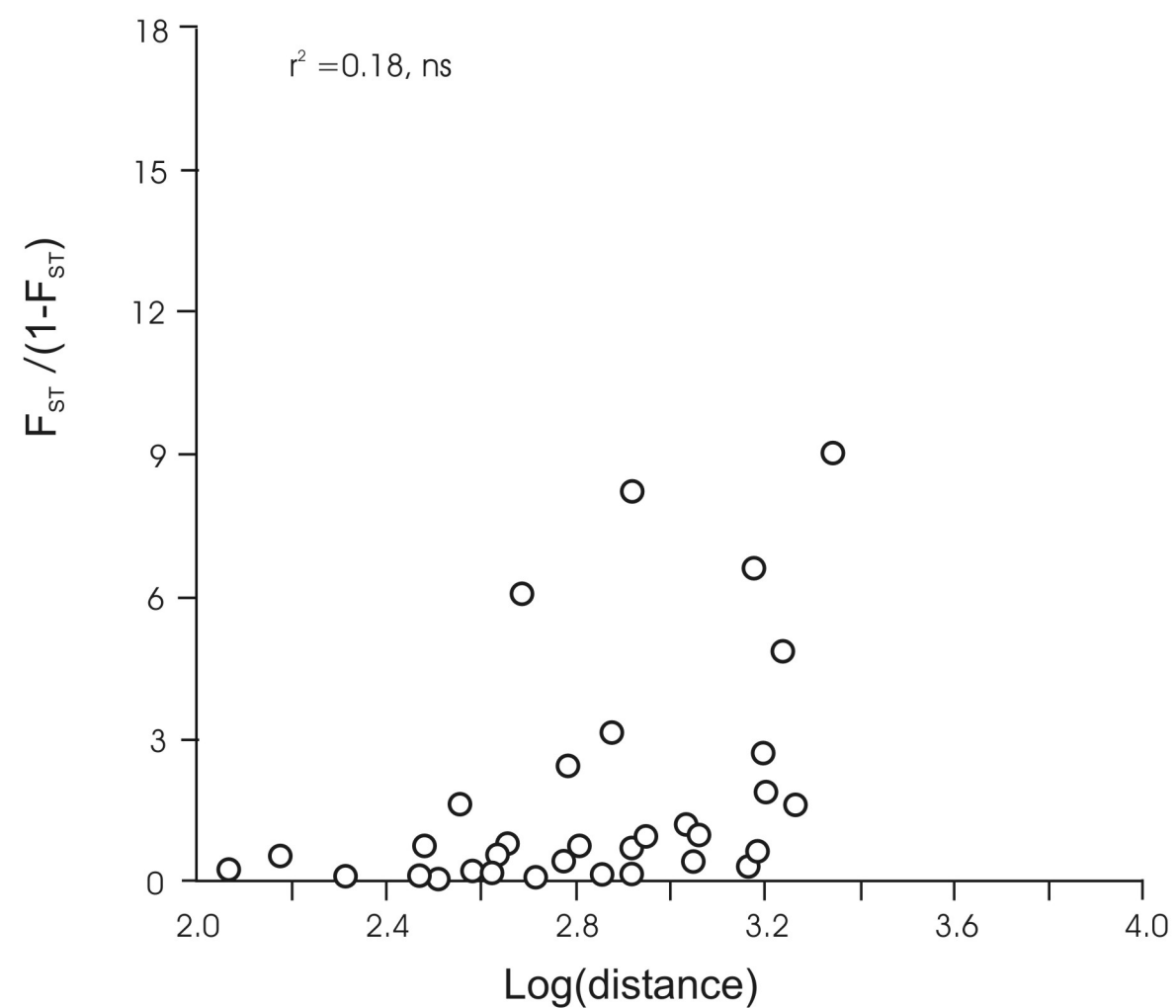

Figure S1

Supplement: S1 Fig — Results of isolation by distance (IBD) analysis of the Eurasian lynx populations. Two upper panels show IBD for microsatellite data for all ten putative populations (A) and excluding Kirov (Russia) (B). Two lower panels show IBD for mtDNA data for all populations (C) and excluding Kirov (Russia) (D). A significant relationship was only obtained for microsatellite data after excluding the Russian population. (PDF) [file pone.0115160.s001.pdf]

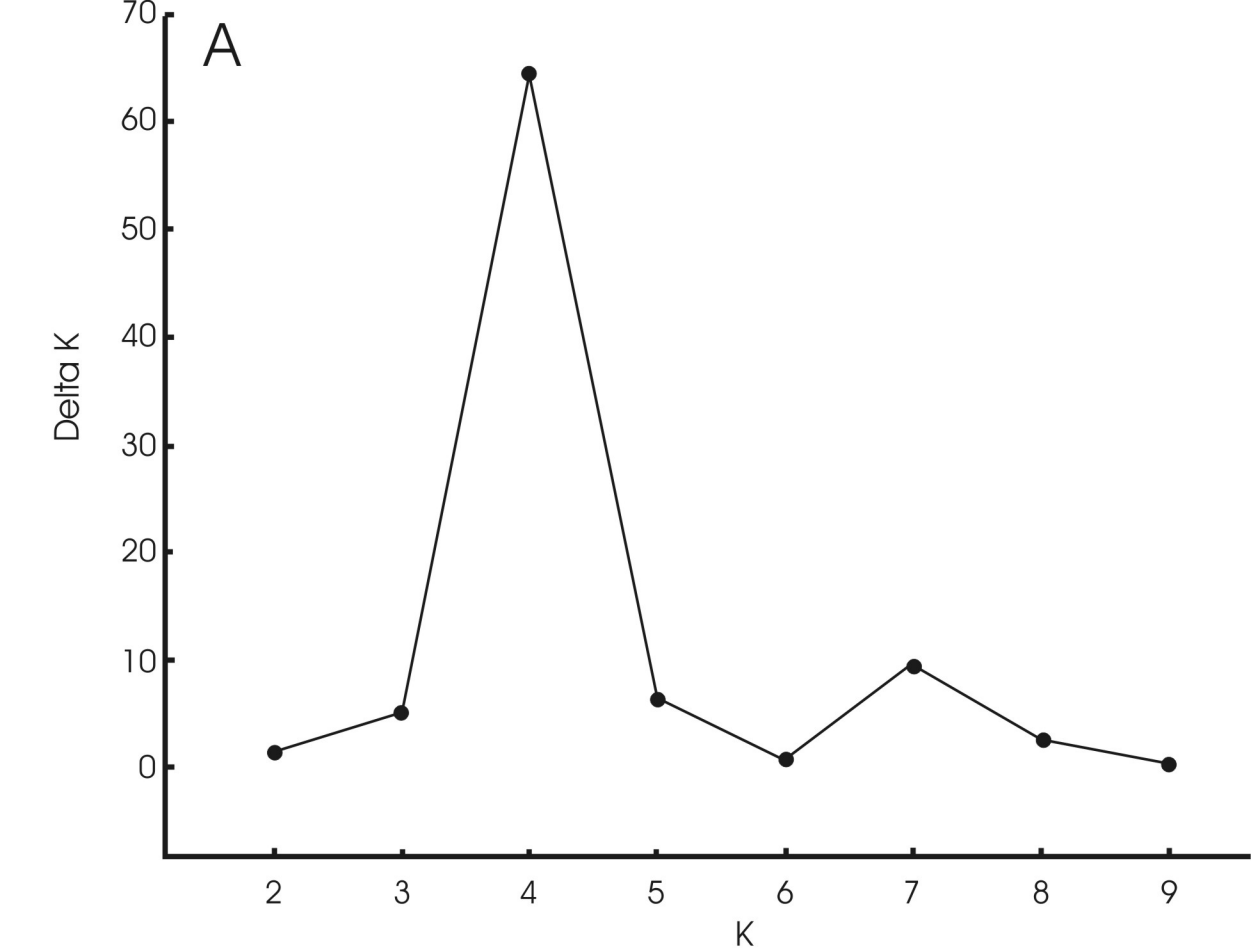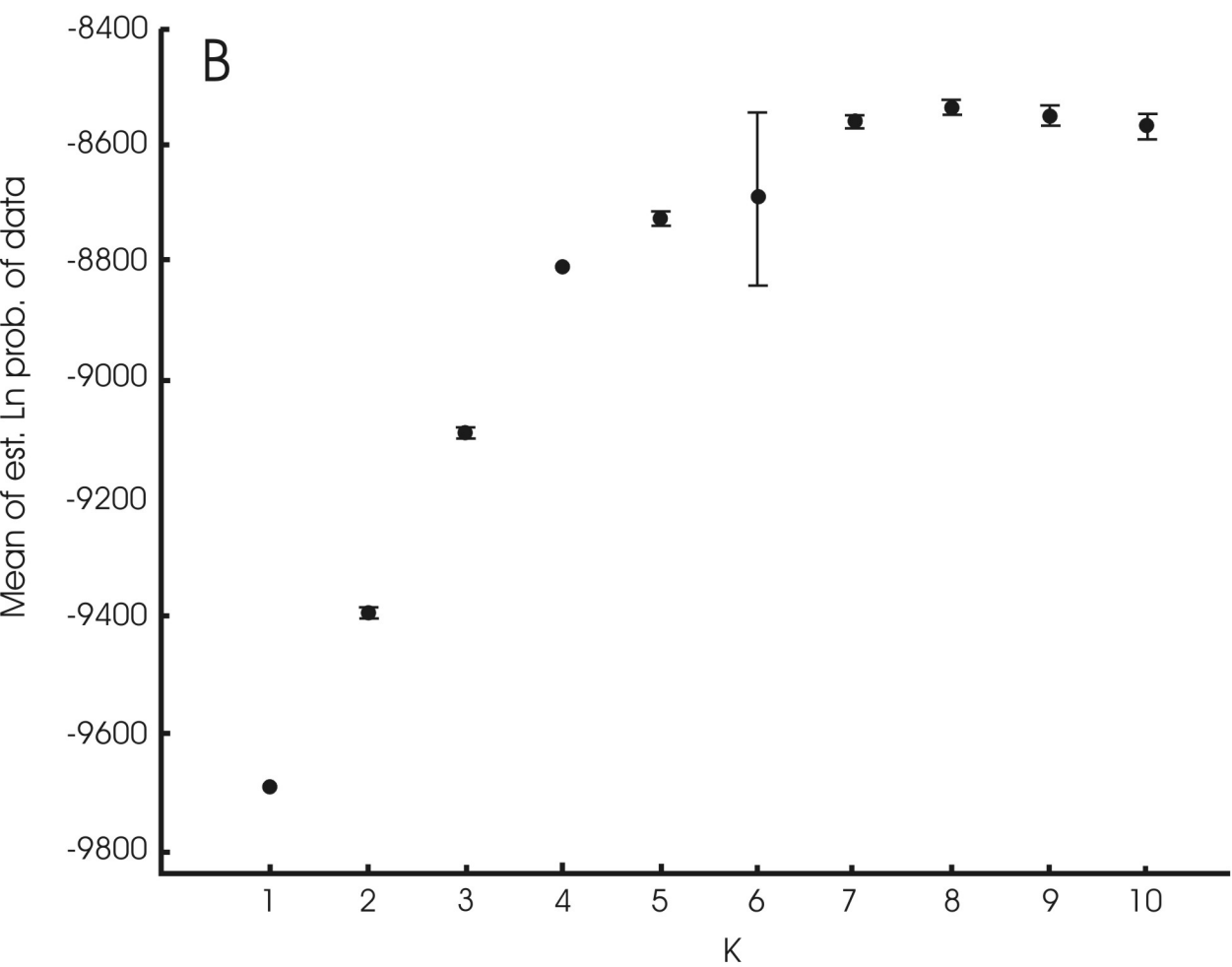

Figure S2

Supplement: S2 Fig — Number of genetic clusters of the Eurasian lynx population in north-eastern Europe. Evaluation of the most likely number of genetic clusters with the ΔK method (A) and the probability logarithm of the data (lnP(D) (B). ΔK clearly suggests that K = 4 is the most likely number of clusters, while lnP(D|K) shows leveling off from K = 4 and the highest likelihood at K = 8. (PDF) [file pone.0115160.s002.pdf]
