# Supplementary material for: Long-Range Gene Flow and the Effects of Climatic and Ecological Factors on Genetic Structuring in a Large, Solitary Carnivore: The Eurasian Lynx
Source: PLoS One. 2014 Dec 31;9(12):e115160. doi: 10.1371/journal.pone.0115160 (PMC4281111; doi:10.1371/journal.pone.0115160)
Supplement: S1 Table — Haplotype frequencies of the mtDNA control region in ten populations of Eurasian lynx Lynx lynx . (DOC) [file pone.0115160.s003.doc]

Table S1. Haplotype frequencies of the mtDNA control region in ten populations of Eurasian lynx *Lynx lynx***.** Populations are grouped into four subgroups as suggested by SAMOVA.

| Haplotype | Norway | Finland | Estonia | Latvia | Lithuania | Belarus | KARPF | Kirov | BPF | Carpathians |
| --- | --- | --- | --- | --- | --- | --- | --- | --- | --- | --- |
|  | SAMOVA groups of populations | | | | | | | | | |
|  | 1 | 2 | | | | | | | 3 | 4 |
| H1 | 100.00 | 51.72 | 50.00 | 27.66 | - | 27.27 | 11.54 | 55.77 | 4.00 | - |
| H2 | - | 13.79 | - | 27.66 | 78.57 | 36.36 | 53.85 | 1.92 | 4.00 | - |
| H3 | - | - | 13.79 | 12.77 | - | - | - | - | - | - |
| H4 | - | - | 13.79 | 17.02 | - | 9.09 | 7.69 | 23.08 | - | 100.00 |
| H6 | - | - | - | - | - | - | - | 3.85 | - | - |
| H8 | - | 34.48 | 3.45 | - | - | - | - | 9.62 | - | - |
| H9 | - | - | 12.07 | 6.38 | - | - | - | 1.92 | - | - |
| H10 | - | - | - | 4.26 | - | - | 26.92 | - | 80.00 | - |
| H11 | - | - | - | - | - | 18.18 | - | - | 12.00 | - |
| H12 | - | - | 6.90 | 4.26 | - | - | - | - | - | - |
| H13 | - | - | - | - | 7.14 | - | - | - | - | - |
| H14 | - | - | - | - | 7.14 | - | - | - | - | - |
| H15 | - | - | - | - | 7.14 | - | - | - | - | - |
| H16 | - | - | - | - | - | 9.09 | - | - | - | - |
| H17 | - | - | - | - | - | - | - | 1.92 | - | - |
| H18 | - | - | - | - | - | - | - | 1.92 | - | - |
