# Supplementary material for: Long-Range Gene Flow and the Effects of Climatic and Ecological Factors on Genetic Structuring in a Large, Solitary Carnivore: The Eurasian Lynx
Source: PLoS One. 2014 Dec 31;9(12):e115160. doi: 10.1371/journal.pone.0115160 (PMC4281111; doi:10.1371/journal.pone.0115160)
Supplement: S2 Table — Pairwise differentiation between Eurasian lynx populations. F ST values based on 12 microsatellites (above diagonal) and F ST values based on the mtDNA control region (below diagonal). Nonsignificant values are given in italics. (DOC) [file pone.0115160.s004.doc]

Table S2. Pairwise differentiation between the Eurasian lynx populations: *F*_ST_ values based on the mtDNA control region. Nonsignificant values are given in italics.

| Population | Norway | Finland | Estonia | Latvia | Lithuania | Belarus | KARPF | BPF | Carpathians |
| --- | --- | --- | --- | --- | --- | --- | --- | --- | --- |
| Finland | 0.37 | - |  |  |  |  |  |  |  |
| Estonia | 0.23 | 0.09 | - |  |  |  |  |  |  |
| Latvia | 0.39 | 0.12 | 0.07 | - |  |  |  |  |  |
| Lithuania | 0.87 | 0.41 | 0.41 | 0.19 | - |  |  |  |  |
| Belarus | 0.62 | 0.13 | 0.12 | *-0.01* | 0.16 | - |  |  |  |
| KARPF | 0.65 | 0.27 | 0.27 | 0.08 | 0.09 | *0.05* | - |  |  |
| BPF | 0.83 | 0.50 | 0.43 | 0.35 | 0.62 | 0.43 | 0.34 | - |  |
| Carpathians | 1.00 | 0.73 | 0.55 | 0.49 | 0.89 | 0.76 | 0.71 | 0.86 | - |
| Kirov | 0.23 | 0.07 | *0.02* | 0.10 | 0.44 | 0.13 | 0.30 | 0.47 | 0.56 |
