# Supplementary material for: Long-Range Gene Flow and the Effects of Climatic and Ecological Factors on Genetic Structuring in a Large, Solitary Carnivore: The Eurasian Lynx
Source: PLoS One. 2014 Dec 31;9(12):e115160. doi: 10.1371/journal.pone.0115160 (PMC4281111; doi:10.1371/journal.pone.0115160)
Supplement: S3 Table — Pairwise differentiation between Eurasian lynx populations. D EST (above diagonal) and R ST (below diagonal) values based on 12 microsatellites. (DOC) [file pone.0115160.s005.doc]

Table S3. Pairwise differentiation between the Eurasian lynx populations: *D*_EST_ (above diagonal) and *R*_ST_ (below diagonal) values based on 12 microsatellites. Nonsignificant values are given in tialics.

| Population | Norway | Finland | Estonia | Latvia | Lithuania | Belarus | KARPF | BPF | Carpathians | Kirov |
| --- | --- | --- | --- | --- | --- | --- | --- | --- | --- | --- |
| Norway | - | 0.22 | 0.16 | 0.16 | 0.17 | 0.14 | 0.14 | 0.22 | 0.31 | 0.17 |
| Finland | 0.15 | - | 0.05 | 0.04 | *0.02* | 0.06 | 0.10 | 0.17 | 0.20 | 0.05 |
| Estonia | 0.15 | 0.07 | - | *0.00* | *0.04* | 0.09 | 0.10 | 0.19 | 0.22 | 0.07 |
| Latvia | 0.15 | 0.05 | *0.01* | - | *0.01* | 0.08 | 0.09 | 0.16 | 0.19 | 0.07 |
| Lithuania | 0.11 | *0.03* | *0.03* | *0.02* | - | 0.06 | 0.04 | 0.07 | 0.16 | 0.07 |
| Belarus | 0.26 | 0.07 | 0.08 | 0.06 | 0.05 | - | 0.10 | 0.21 | 0.19 | 0.07 |
| KARPF | 0.23 | 0.07 | 0.10 | 0.08 | 0.02 | 0.08 | - | *0.01* | 0.23 | 0.12 |
| BPF | 0.24 | 0.14 | 0.19 | 0.17 | 0.09 | 0.18 | *0.00* | - | 0.32 | 0.20 |
| Carpathians | 0.21 | 0.05 | 0.13 | 0.09 | 0.05 | 0.11 | 0.14 | 0.23 | - | 0.16 |
| Kirov | 0.16 | 0.07 | 0.04 | 0.06 | *0.00* | 0.05 | *0.02* | 0.08 | 0.15 | - |
