# Supplementary material for: Long-Range Gene Flow and the Effects of Climatic and Ecological Factors on Genetic Structuring in a Large, Solitary Carnivore: The Eurasian Lynx
Source: PLoS One. 2014 Dec 31;9(12):e115160. doi: 10.1371/journal.pone.0115160 (PMC4281111; doi:10.1371/journal.pone.0115160)
Supplement: S4 Table — Habitat type, climate and lynx diet composition of the ten lynx populations studied. * References used for data on lynx diet: Norway [84]; Finland: [85]; Estonia and Latvia: [86]; Belarus: [87]; Poland (KARPF): [88]; Poland (BPF): [27]; Carpathians: [89]; Russia (Kirov): [90]. No data were available for Lithuania – for the computations we arbitrarily assumed the same prey composition as in Latvia due to very close geographical proximity. The data were recalculated from original sources considering the relative share of only key prey that occurred in all populations. (DOC) [file pone.0115160.s006.doc]

Table S4. Habitat type, climate and lynx diet composition of the ten lynx populations studied.

| Population | Climatic variables | | | | Share (%) of main prey species in lynx diet* | | | | |
| --- | --- | --- | --- | --- | --- | --- | --- | --- | --- |
|  | Mean temperature January °C | Days with snow cover | Snow cover depth | NAO index | European roe deer  (*Capreolus capreolus*) | Red deer  (*Cervus elaphus*) | Reindeer  (*Rangifer tarandus*) | Hares  (*Lepus capensis*, *L. timidus*) | Grouse  (*Tetraonidae*) |
| Norway | -8.0 | 185 | 40 | -5.0 | 36.2 | 0.3 | 30.8 | 25.3 | 7.4 |
| Finland | -10.0 | 175 | 35 | -4.0 | 0 | 0 | 0 | 86.2 | 12.8 |
| Estonia | -8.0 | 120 | 20 | -2.0 | 56.4 | 1.9 | 0 | 37.3 | 4.4 |
| Latvia | -7.0 | 90 | 16 | -1.0 | 88.9 | 0 | 0 | 11.1 | 0 |
| Lithuania | -4.0 | 80 | 11 | 0.0 | - | - | - | - | - |
| Belarus | -4.0 | 110 | 10 | 1.0 | 41.5 | 0 | 0 | 47.3 | 11.2 |
| Poland (KARPF) | -3.5 | 65 | 12 | 1.0 | 66 | 22 | 0 | 12 | 0 |
| Poland (BPF) | -4.5 | 80 | 10 | 1.0 | 65.4 | 23.5 | 0 | 9.3 | 1.8 |
| Carpathians | -4.0 | 50 | 12 | 3.0 | 72.3 | 17 | 0 | 4.3 | 6.4 |
| Russia (Kirov) | -15.0 | 180 | 41 | -3.0 | 0 | 0 | 0 | 82.2 | 17.8 |

* References used for data on lynx diet: Norway [84]; Finland: [85]; Estonia and Latvia: [86]; Belarus: [87]; Poland (KARPF): [88]; Poland (BPF): [27]; Carpathians: [89]; Russia (Kirov): [90]. No data were available for Lithuania – for the computations we arbitrarily assumed the same prey composition as in Latvia due to very close geographical proximity. The data were recalculated from original sources considering the relative share of only key prey that occurred in all populations.
