# Supplementary material for: Long-Range Gene Flow and the Effects of Climatic and Ecological Factors on Genetic Structuring in a Large, Solitary Carnivore: The Eurasian Lynx
Source: PLoS One. 2014 Dec 31;9(12):e115160. doi: 10.1371/journal.pone.0115160 (PMC4281111; doi:10.1371/journal.pone.0115160)

**Appendix S2** Procedures adopted in the Approximate Bayesian Computations (DIYABC 2.0.4 software) and the list of all scenarios tested.

**Climatic and environmental factors along with genetic drift affect population differentiation and gene flow in the large solitary carnivore - the Eurasian lynx**

M. Ratkiewicz, M. Matosiuk, A. P. Saveljev, V. Sidorovich, J. Ozolins, P. Männil, L. Balciauskas, I. Kojola, H. Okarma, R. Kowalczyk, K. Schmidt

We applied Approximate Bayesian Computation (ABC; using the program DIYABC 2.0.4; [1]) to estimate the relative likelihood of possible scenarios for the lynx population histories. We conducted analyses separately for microsatellite and mitochondrial DNA data. Combining microsatellite and mtDNA data into single analysis as suggested by Cornuet et al. [2] appeared unsuccessful as the estimation of prior distribution of parameters showed a lack of correspondence between simulated and the observed data sets. We tested nine possible scenarios of lynx population histories assuming a subdivision into four subpopulations: 1) Norway, 2) BPF-KARPF, 3) Baltic and 4) Russia-Finland, suggested by the STUCTURE analysis. However, we decided to distinguish the fifth group - the Carpathian population due to its separate character suggested by SAMOVA.

We ran the analyses in two settings: 1) we tested all nine scenarios against each other to select the best supported one and 2) following the procedure suggested by Pedreschi et al. [3] we tested three groups with three different scenarios each to select the best scenario per group and then we tested the best scenarios against each other in the final analysis. All the predicted nine scenarios that were analyzed both with microsatellite and mtDNA data are illustrated in Fig. S1.

We set the effective population sizes (Ne) from 10 to 10000. Each competing scenario was given equal prior probability. For the microsatellite data, we accepted the default mutation rate model prior distributions suggested in the software. For the mitochondrial dataset we assumed the mean mutation rate of 5.35×10^−7^ substitutions/site/year with SD = 2.28×10^−7^ substitutions estimated for African lions [4]. We set the number of simulated data sets at 500000.

We ran computations for microsatellites to measure the similarity between the observed and simulated datasets with following summary statistics: mean number of alleles, mean gene diversity and mean allele size variance across loci (for single and two sample population estimates); F*_ST_* between two samples, mean index of classification and (δμ)^2^ distance between two samples (for two sample population estimates). For mtDNA data we ran simulations with the number of haplotypes, number of segregating sites, mean and variance of the number of pairwise differences and Tajima's D statistics (for single sample population estimates); number of haplotypes and segregating sites in the pooled sample, mean of within and between sample pairwise differences, and F_ST_ between two samples (for two sample population estimates). We checked model validity by conducting a PCA of summary statistics for each scenario, comparing the distribution of prior and posterior estimates of param­eters, and real data. The validity of the model is confirmed if the point representing real data sets is surrounded by a cloud of points representing posterior estimates (Figs. S2, S4, S6, S8, S11, S13, S15, S17). We compared the relative likelihoods of the scenarios by logistic regression approach with 2% subsets of the closest simulated data (Figs. S3, S5, S7, S9, S10, S12, S14, S16, S18, S19).

**References:**

1. Cornuet J-M, Pudlo P, Veyssier J,. Dehne-Garcia A, Gautier M, Leblois R, Marin J-M, Estoup A (2014) DIYABC v2.0: a software to make approximate Bayesian computation inferences about population history using single nucleotide polymorphism, DNA sequence and microsatellite data. Bioinformatics 30: 1187-1189.

2. Cornuet, J.-M., V. Ravigne, and A. Estoup. 2010. Inference on population history and model checking using DNA sequence and microsatellite data with the software DIYABC (v1.0). BMC Bioinformatics 11:401.

3. Pedreschi, D., M. Kelly-Quinn, J. Caffrey, M. O'Grady, and S. Mariani. 2014. Genetic structure of pike (Esox lucius) reveals a complex and previously unrecognized colonization history of Ireland. Journal of Biogeography 41:548-560.

4. Barnett R, Yamaguchi N, Shapiro B, Ho S, Barnes I, Sabin R, Werdelin L, Cuisin J, Larson G (2014) Revealing the maternal demographic history of Panthera leo using ancient DNA and a spatially explicit genealogical analysis. BMC Evol Biol 14:70.

**Figure S1.** Set of nine possible scenarios of Eurasian lynx colonization patterns in Europe analyzed with Approximate Bayesian Computation with microsatellite and mtDNA datasets (DIYABC 2.0.4 software, Cornuet et al. 2014). Different colors indicate different population sizes.


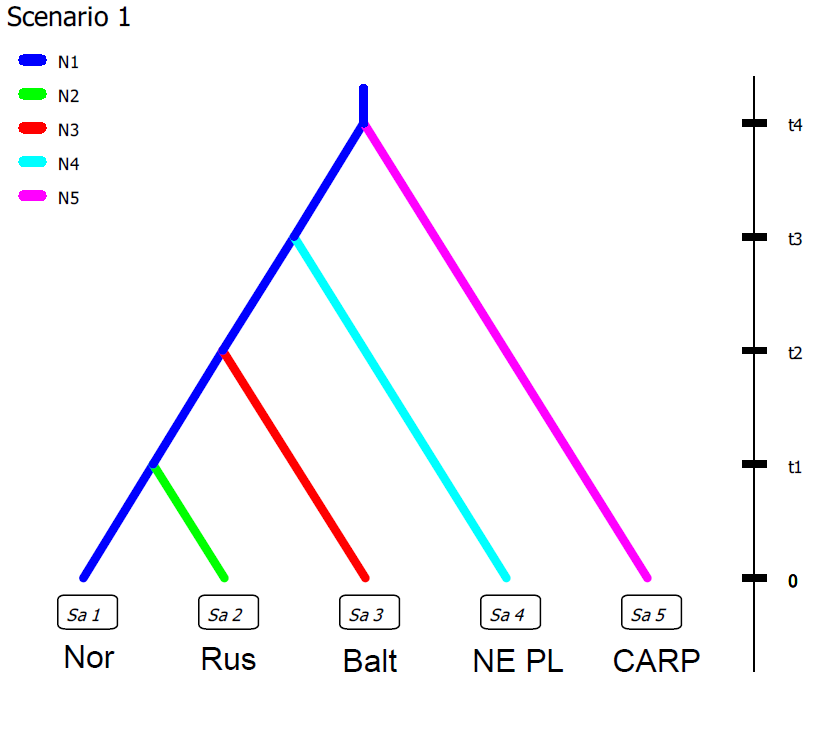

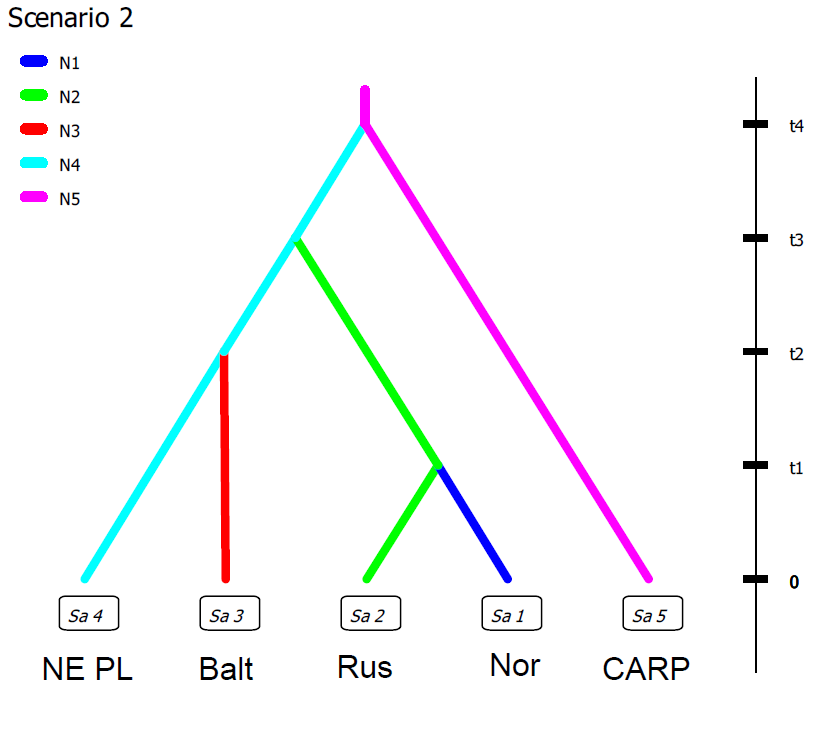

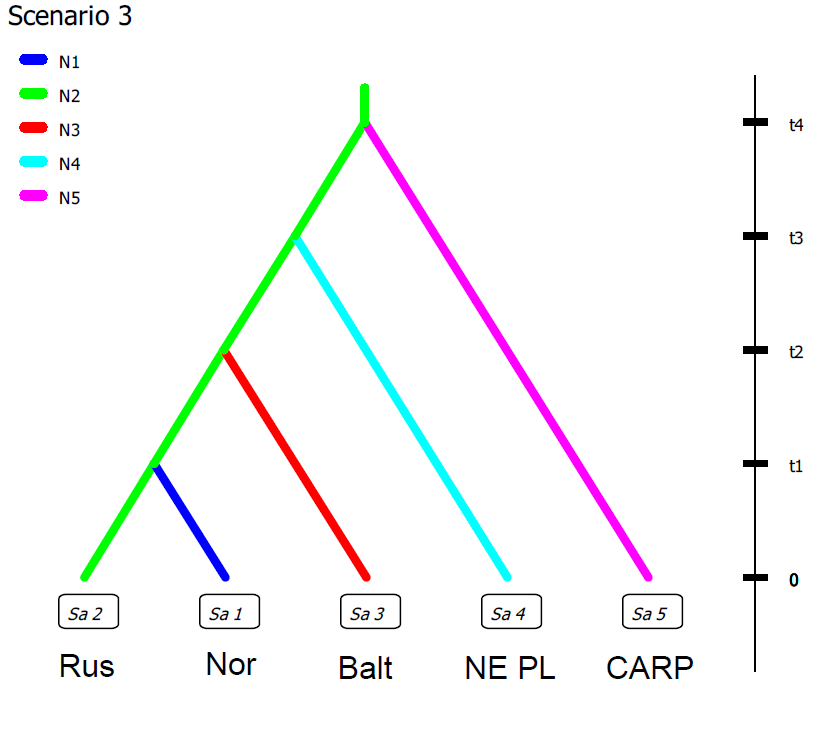


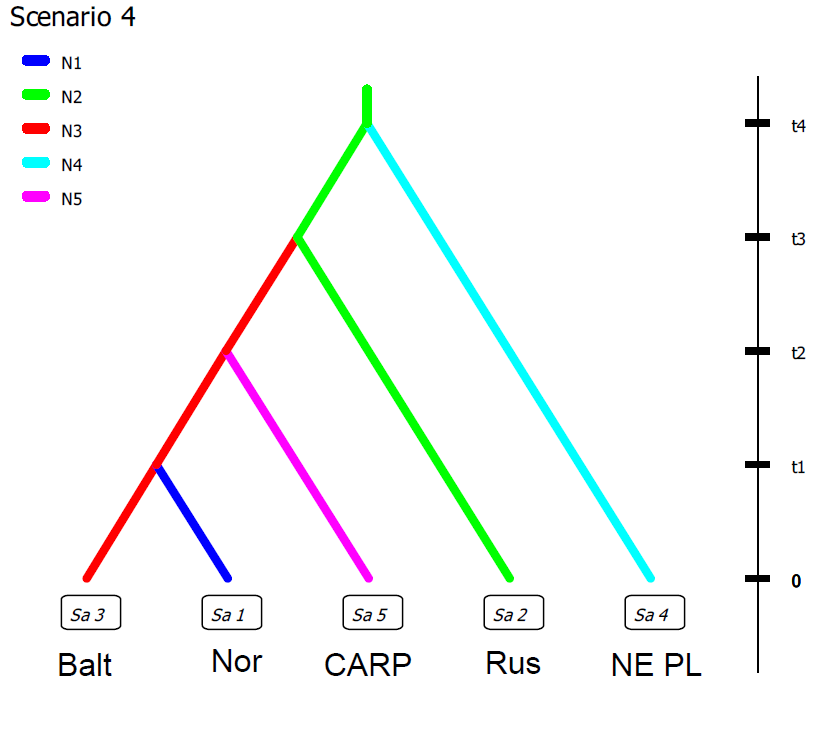

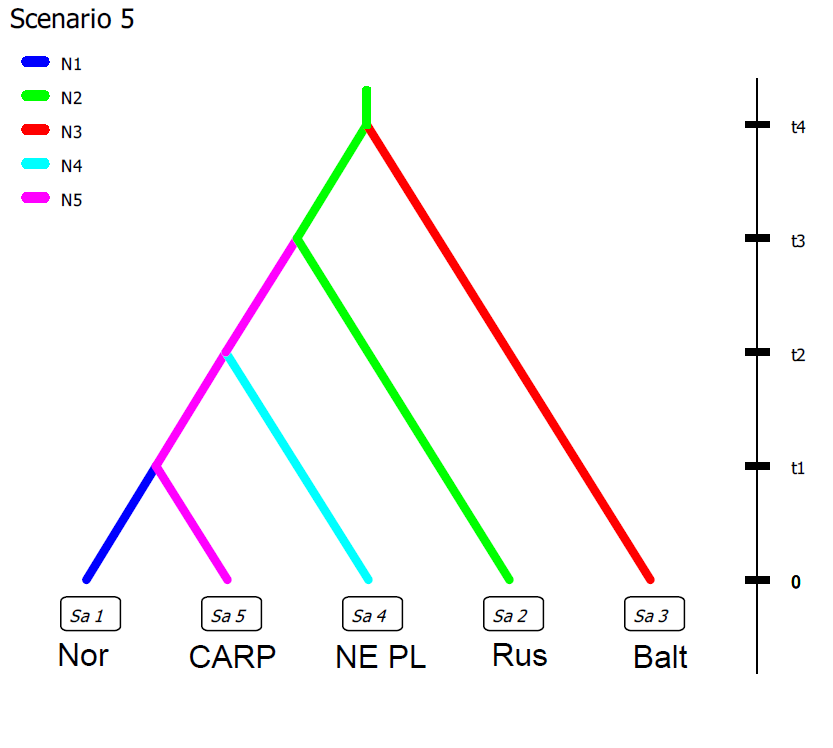

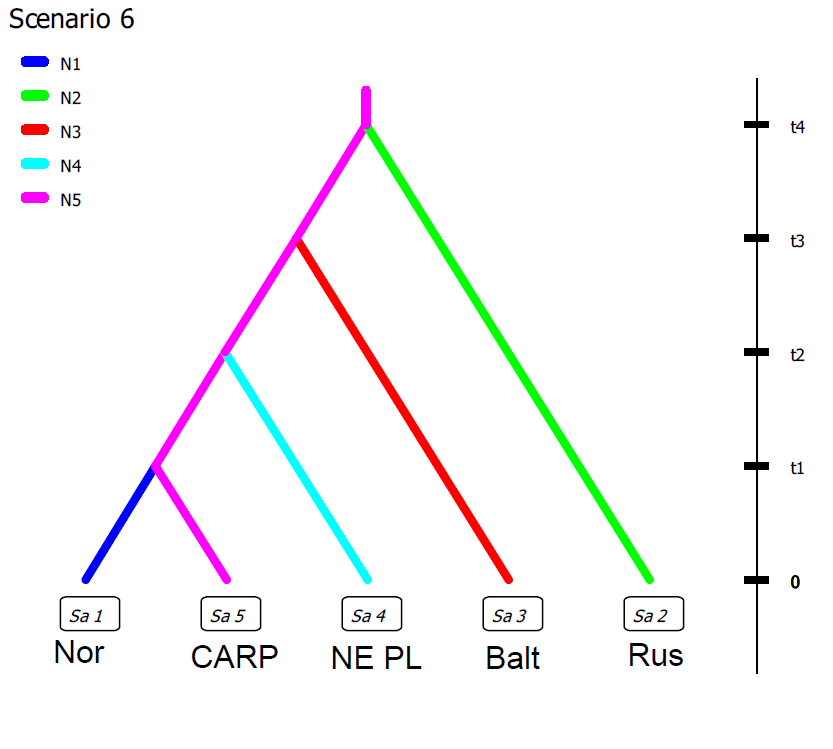


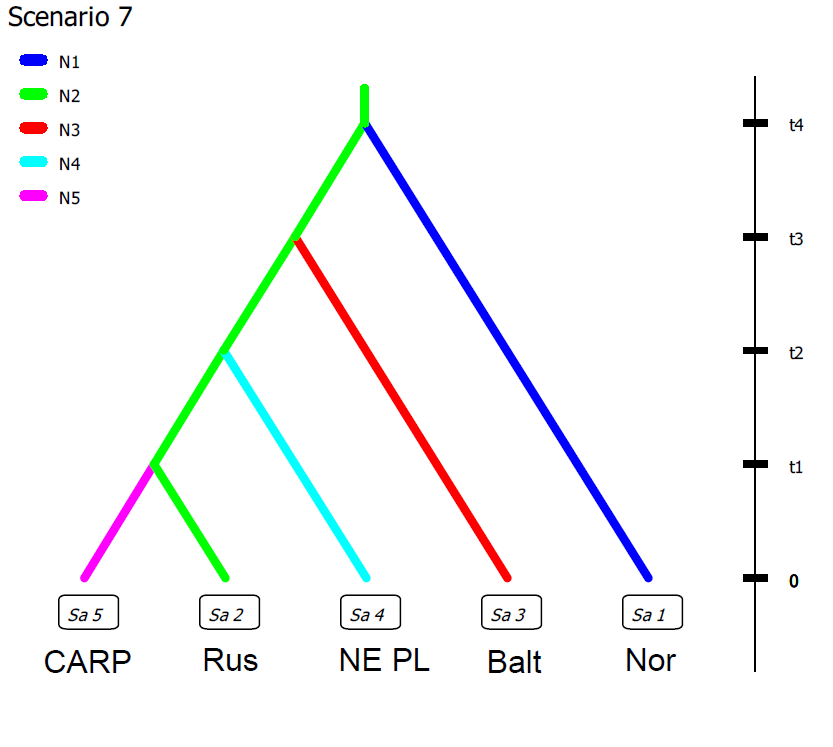

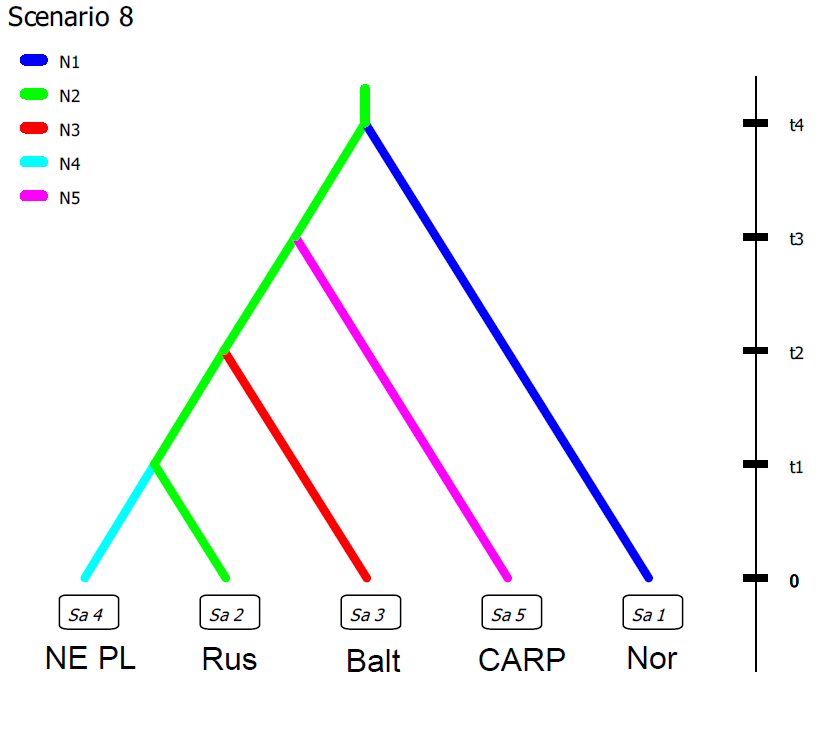

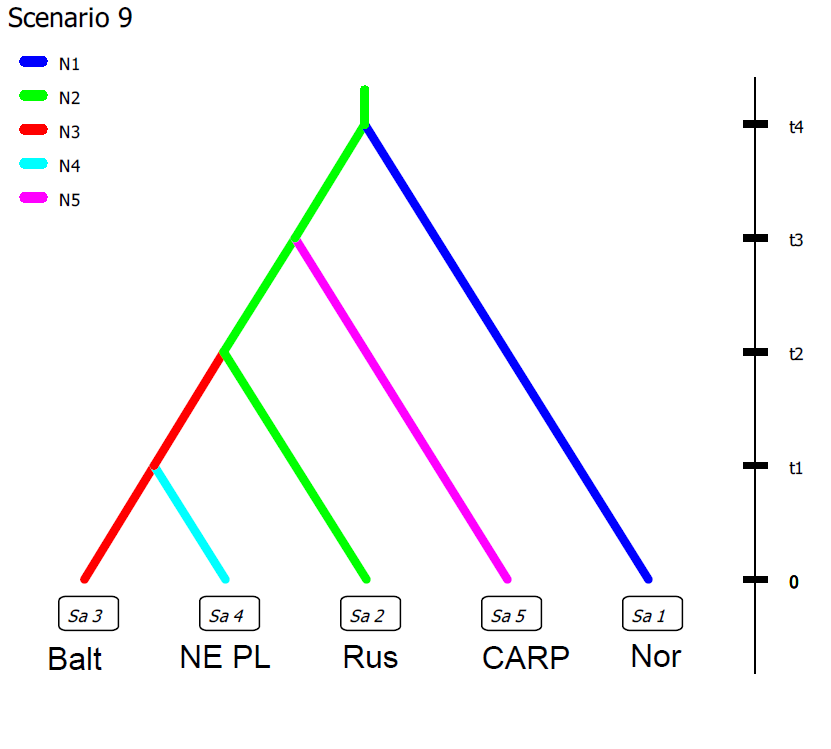


**ABC analyses based on 12 microsatellite loci**

*Group 1 - Scenarios 1, 2, 3*

**Figure S2.** Model checking to measure the discrepancy between the parameters’ posterior combination and real datasets in scenarios 1, 2, and 3.


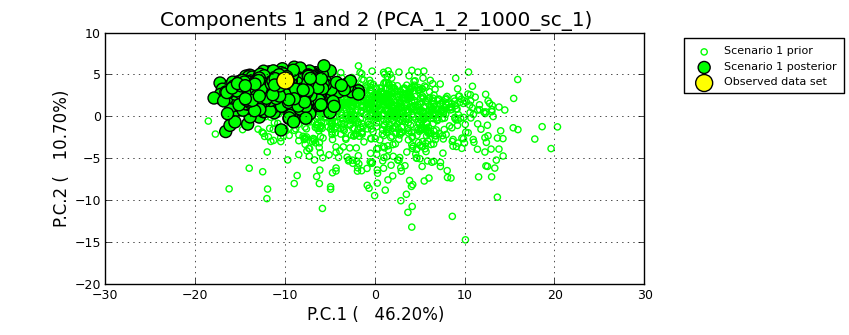

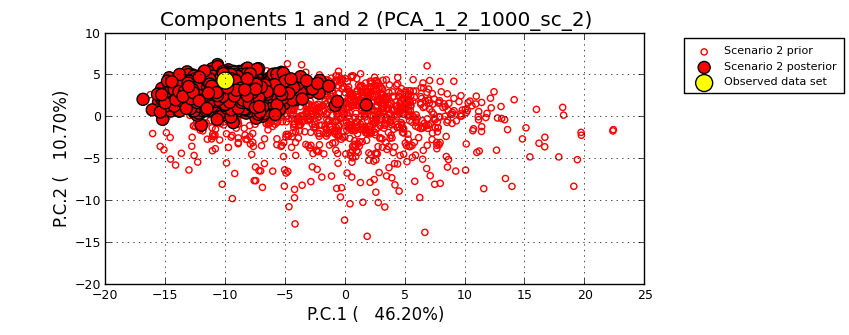


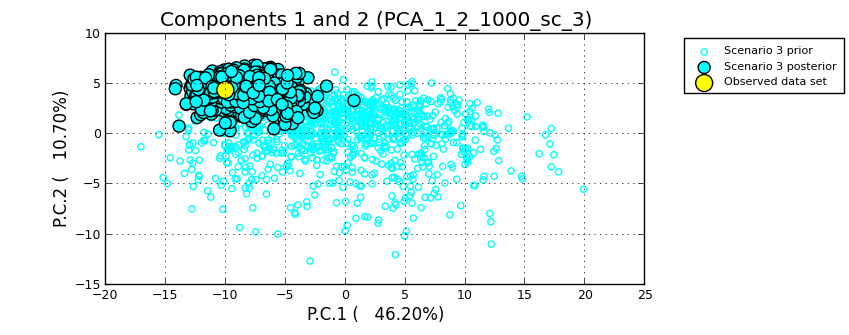


**Figure S3.** Comparison of scenarios 1, 2 and 3 for Eurasian lynx population histories. **Scenario 2** has the highest posterior probability.


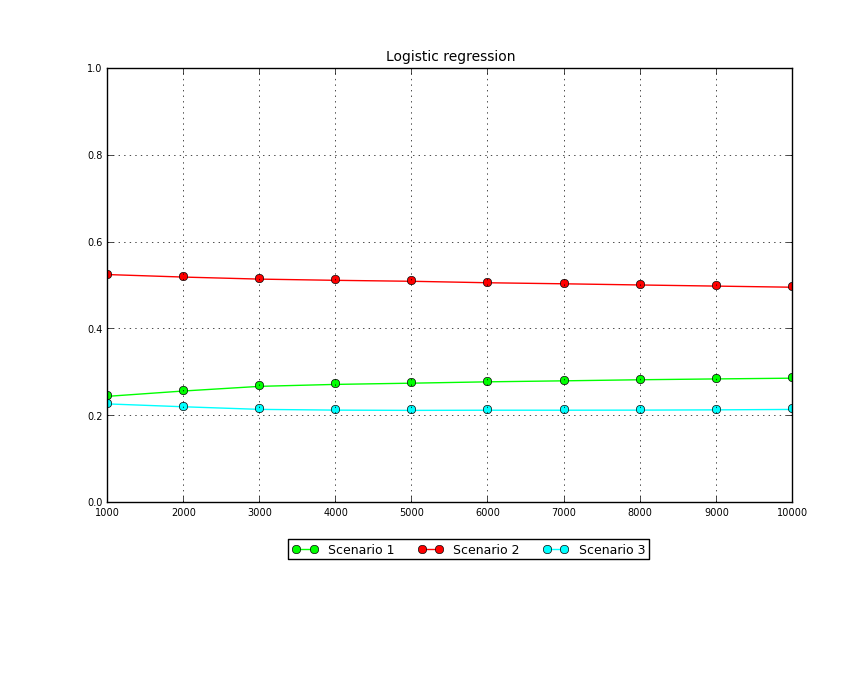


*Group 2 - Scenarios 4, 5, 6*

**Figure S4.** Model checking to measure the discrepancy between the parameters’ posterior combination and real datasets in scenarios 4, 5 and 6.


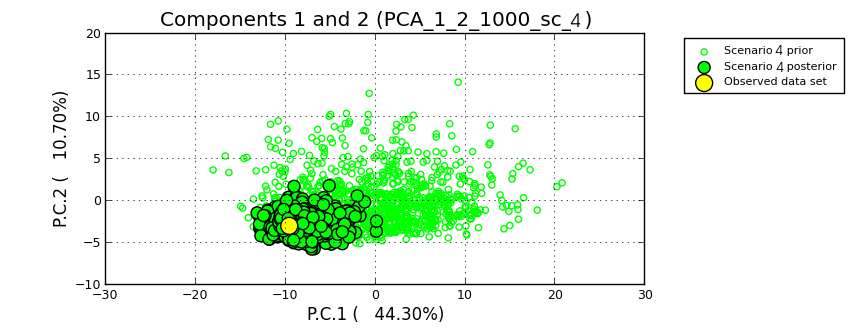


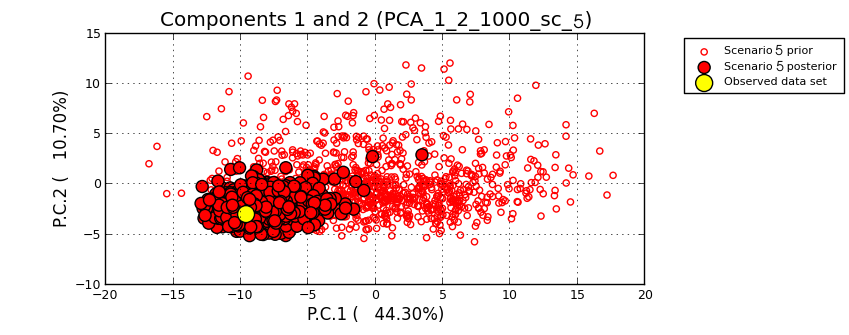


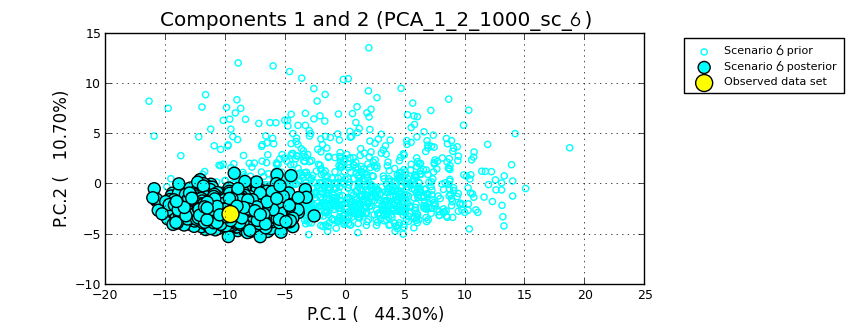


**Figure S5.** Comparison of scenarios 4, 5 and 6 for Eurasian lynx population histories. **Scenario 6** has the highest posterior probability.


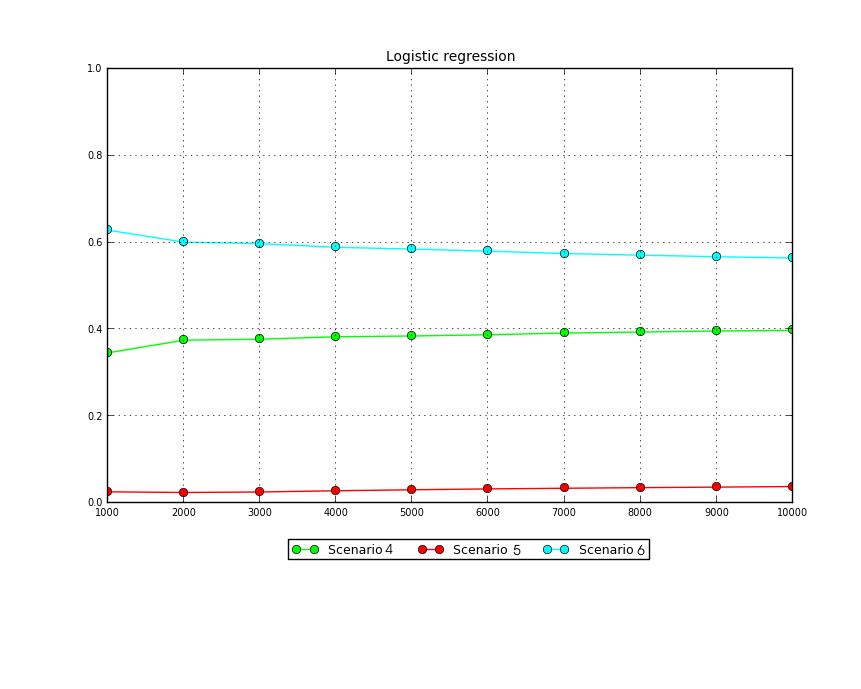


*Group 3 - Scenarios 7, 8 9*

**Figure S6.** Model checking to measure the discrepancy between the parameters’ posterior combination and real datasets in scenarios 7, 8 and 9.


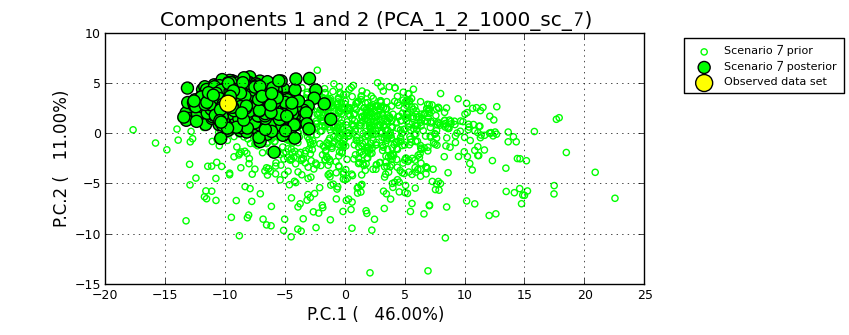

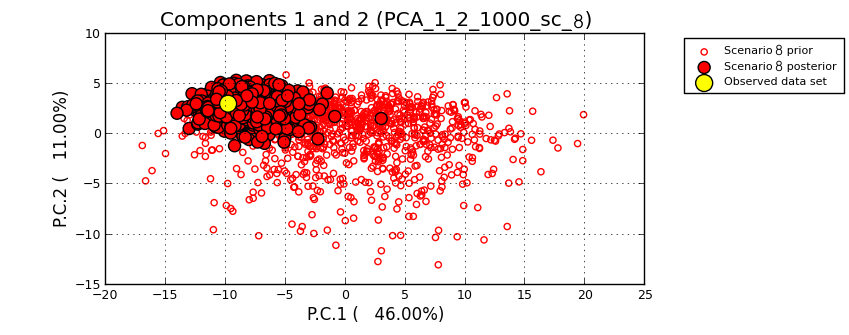

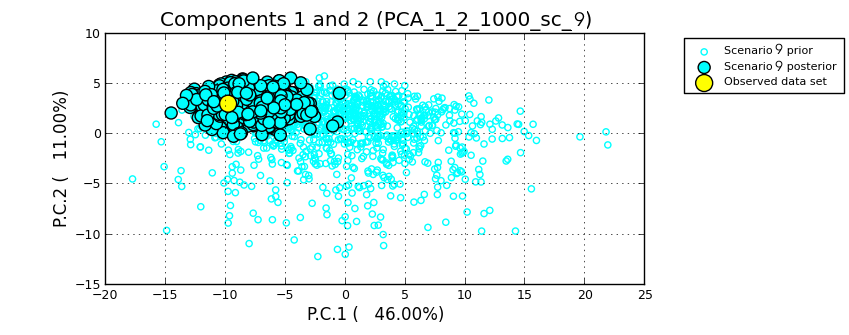


**Figure S7.** Comparison of scenarios 7, 8 and 9 for Eurasian lynx population histories. **Scenario 9** has the highest posterior probability.


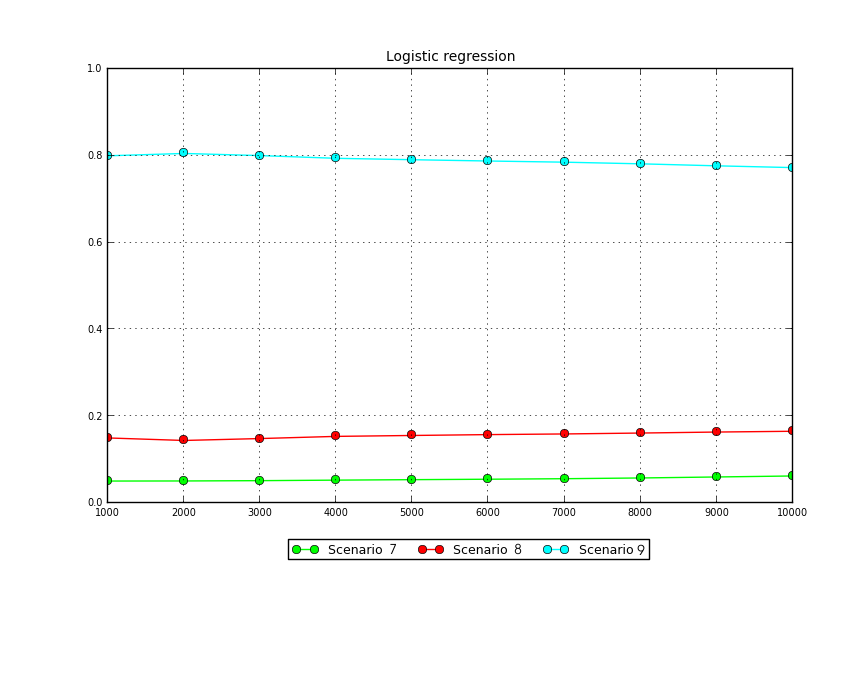


Final group – comparison of best-supported scenarios in groups 1-3 (Scenarios 2, 6, 9).

**Figure S8.** Model checking to measure the discrepancy between the parameters’ posterior combination and real datasets in scenarios 2, 6 and 9.


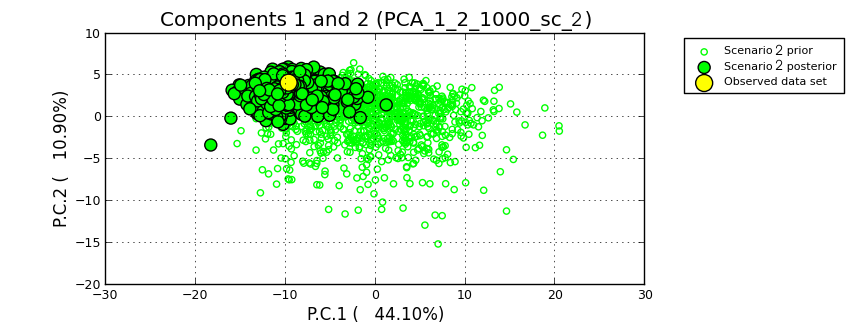


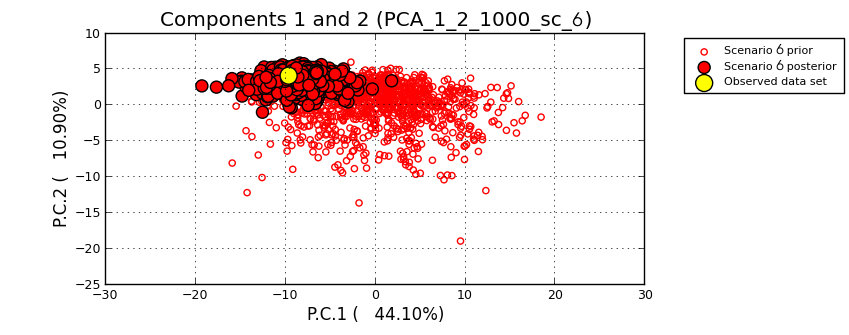


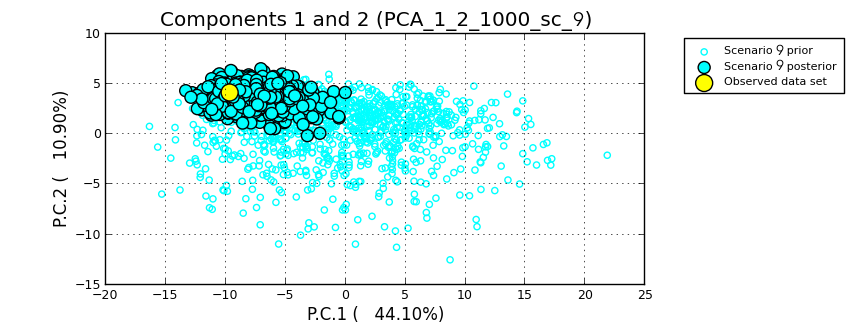


**Figure S9.** Comparison of best supported scenarios (2, 6 and 9) in groups 1, 2 and 3 for Eurasian lynx population histories. **Scenario 2** has the highest posterior probability.


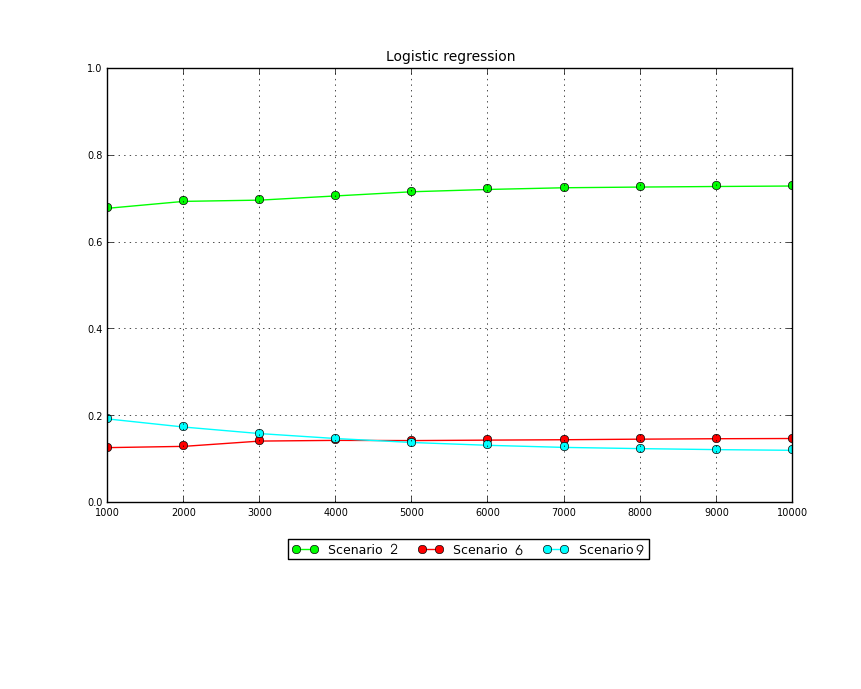


**Figure S10.** Comparison of all scenarios (1 - 9) against each other for Eurasian lynx population histories. **Scenario 2** has the highest posterior probability.


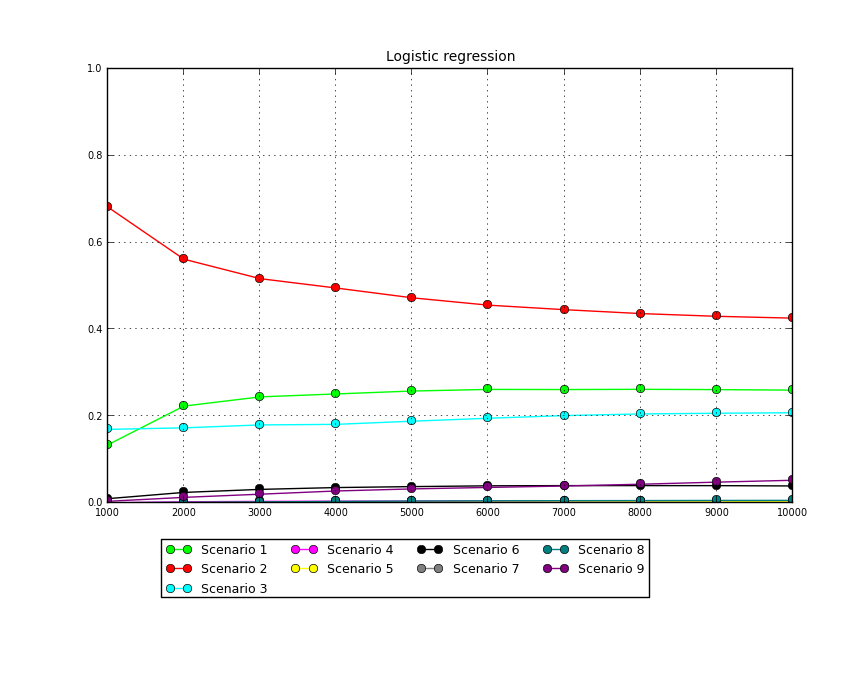


**ABC analyses based on mitochondrial DNA control region**

*Group 1 - Scenarios 1, 2, 3*

**Figure S11.** Model checking to measure the discrepancy between the parameters’ posterior combination and real datasets in scenarios 1, 2, and 3.


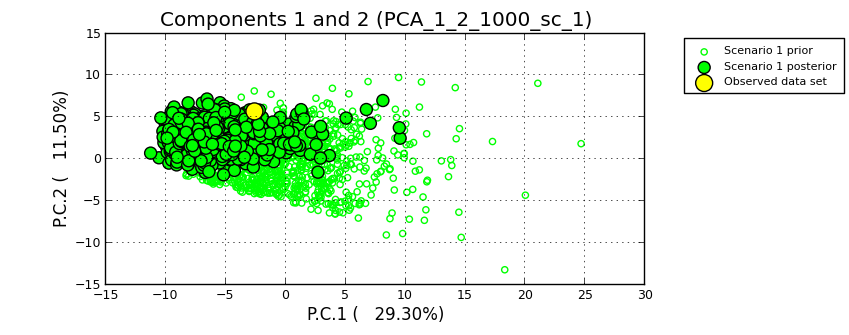

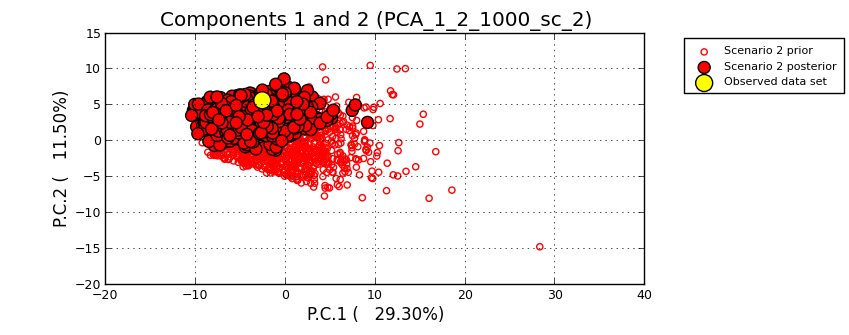


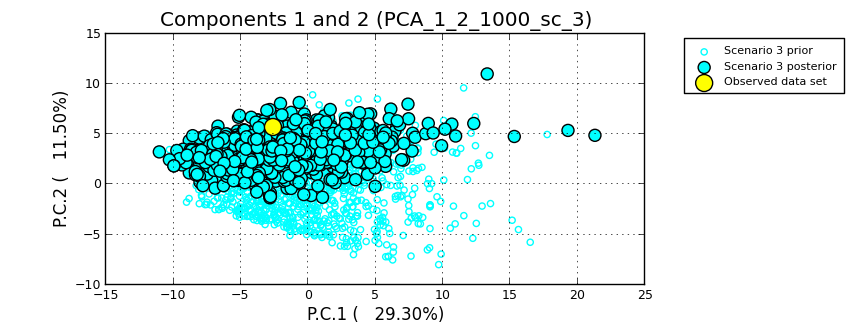


**Figure S12.** Comparison of scenarios 1, 2 and 3 for Eurasian lynx population histories. **Scenario 2** has the highest posterior probability.


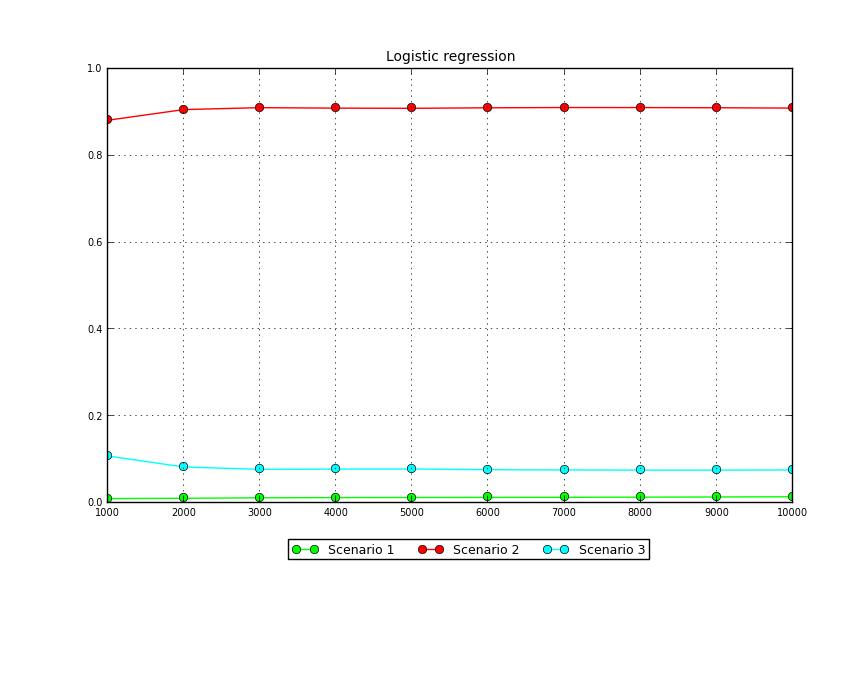


*Group 2 - Scenarios 4, 5, 6*

**Figure S13.** Model checking to measure the discrepancy between the parameters’ posterior combination and real datasets in scenarios 4, 5 and 6.


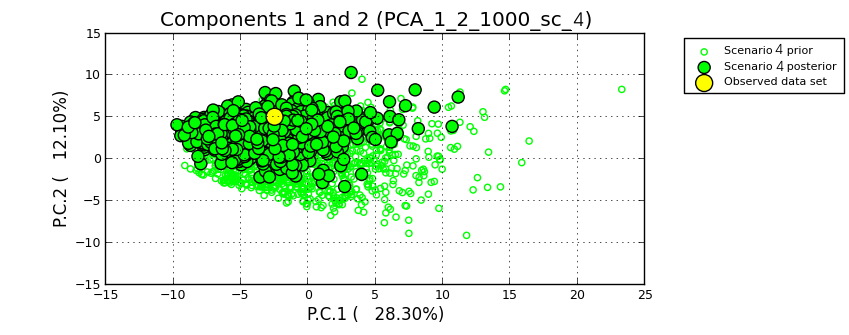

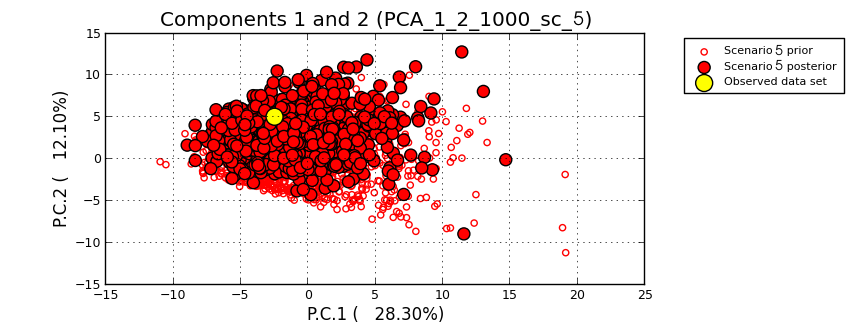


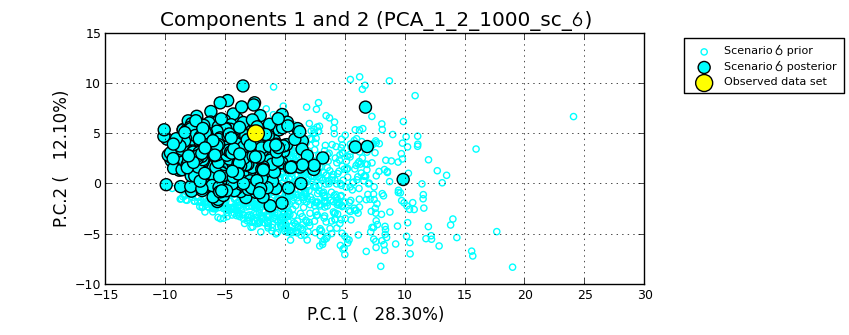


**Figure S14.** Comparison of scenarios 4, 5 and 6 for Eurasian lynx population histories. **Scenarios 4 and 6** have the highest posterior probability.


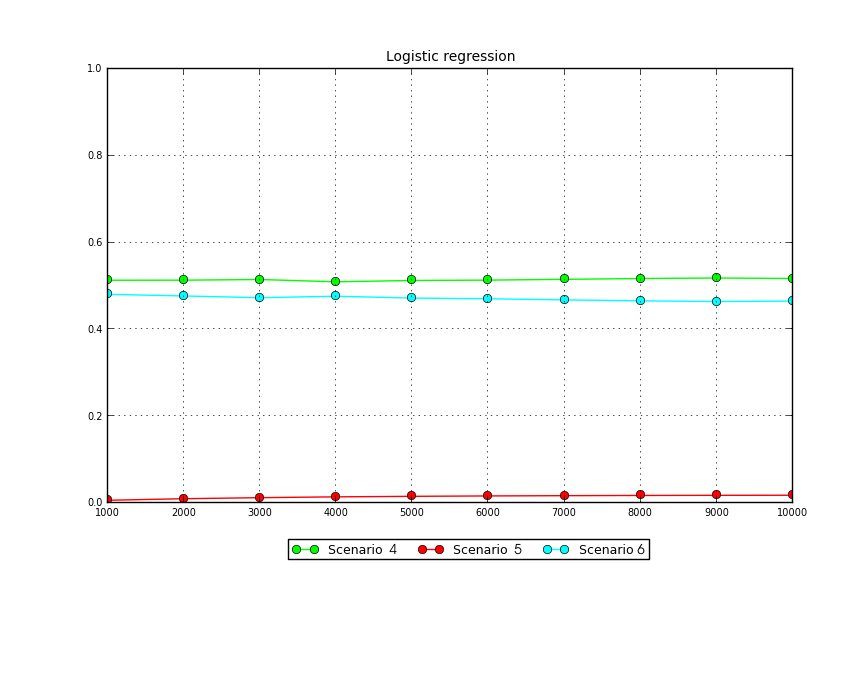


*Group 3 - Scenarios 7, 8 9*

**Figure S15.** Model checking to measure the discrepancy between the parameters’ posterior combination and real datasets in scenarios 7, 8 and 9.


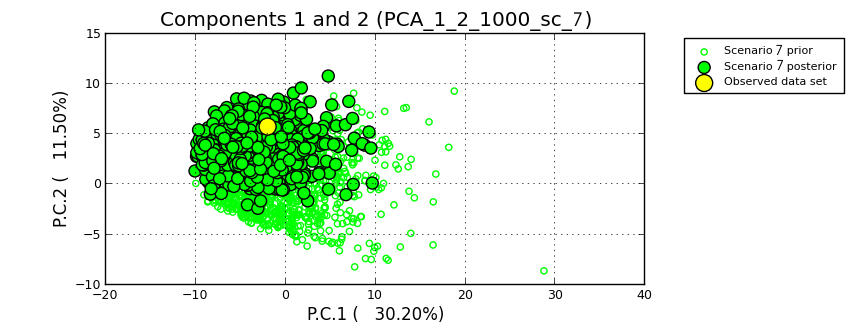

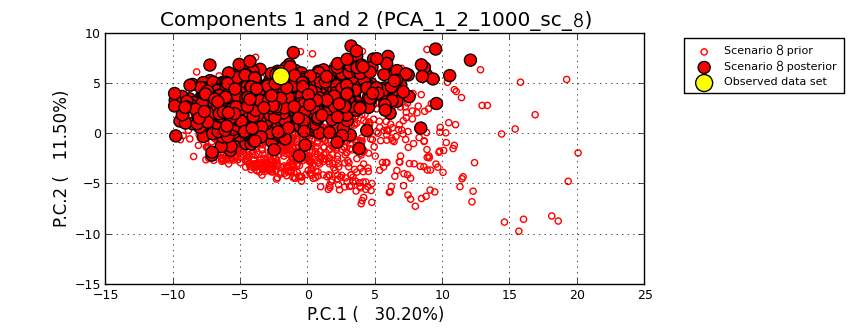

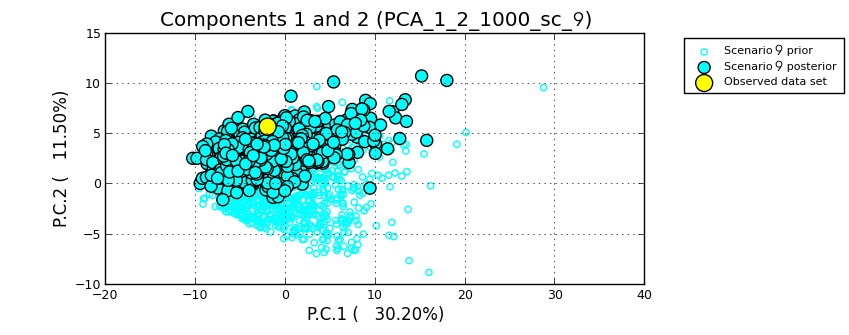


**Figure S16.** Comparison of scenarios 7, 8 and 9 for Eurasian lynx population histories. **Scenario 9** has the highest posterior probability.


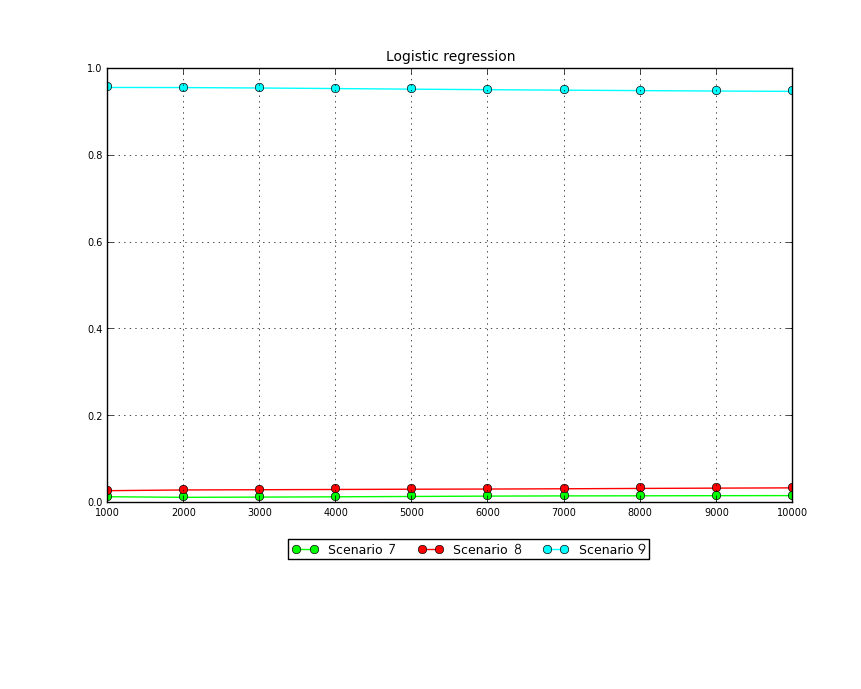


Final group – comparison of best-supported scenarios in groups 1-3 (Scenarios 2, 4, 6, 9).

**Figure S17.** Model checking to measure the discrepancy between the parameters’ posterior combination and real datasets in scenarios 2, 4, 6 and 9.


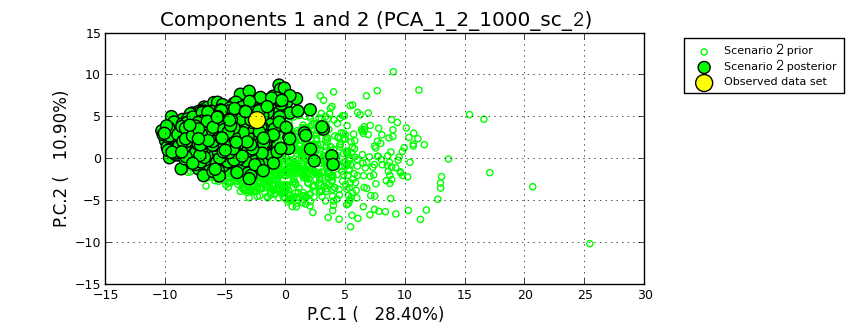

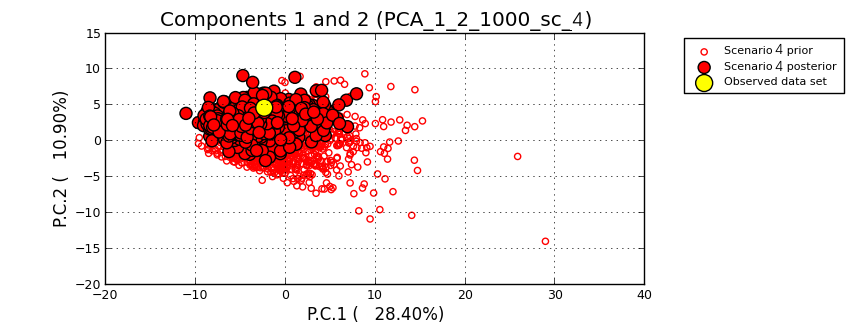

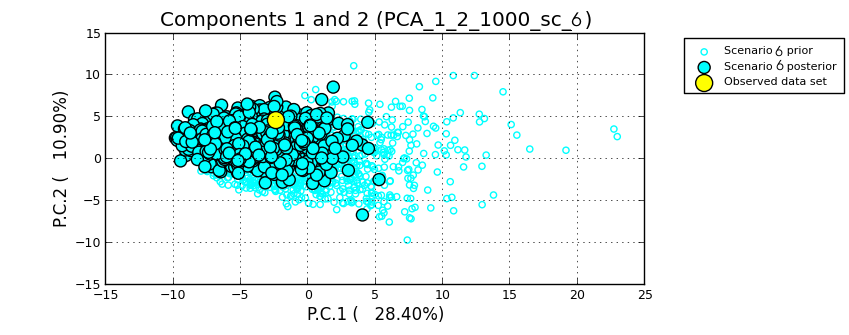


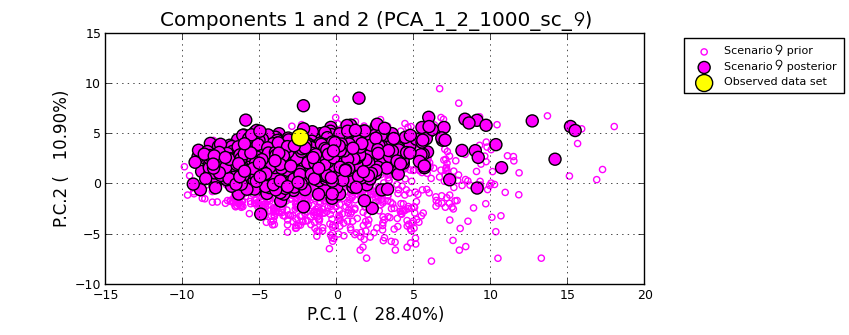


**Figure S18.** Comparison of best supported scenarios (2, 4, 6 and 9) in groups 1, 2 and 3 for Eurasian lynx population histories. **Scenario 9** has the highest posterior probability.


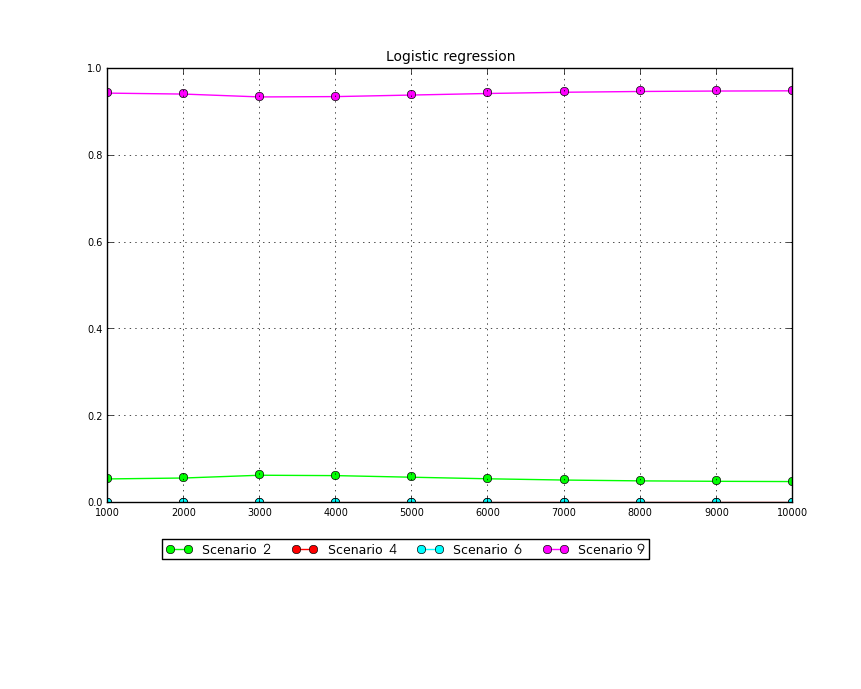


**Figure S19.** Comparison of all scenarios (1 - 9) against each other for Eurasian lynx population histories. **Scenario 9** has the highest posterior probability.


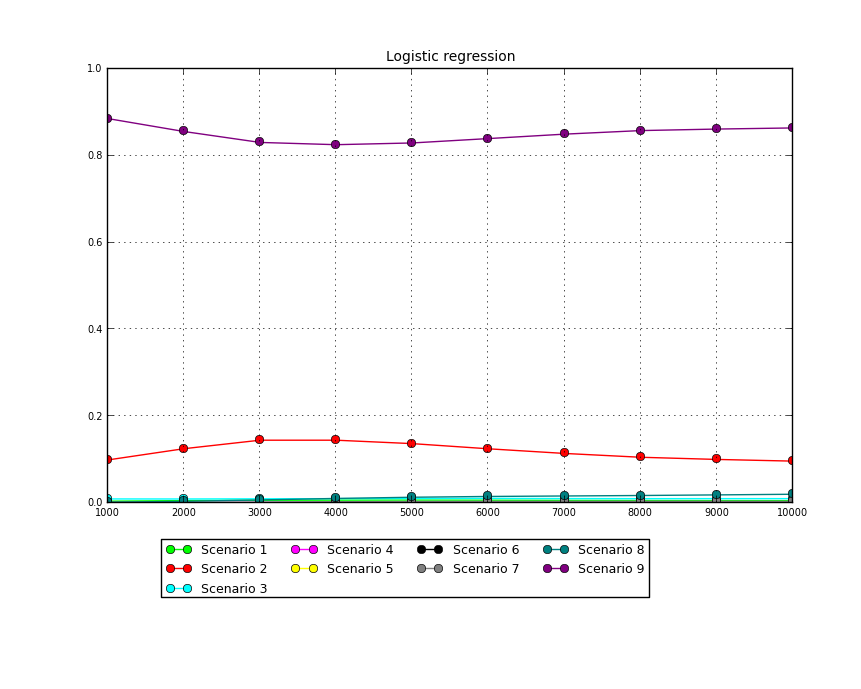

Supplement: S2 Appendix — Procedures adopted in the Approximate Bayesian Computations (DIYABC 2.0.4 software) and the list of all scenarios tested. (DOC) [file pone.0115160.s008.doc]
